# Hair Steroid Signatures of Cooperative Breeding in Male and Female Common Marmosets (*Callithrix jacchus*)

**DOI:** 10.1007/s10764-026-00568-9

**Published:** 2026-05-23

**Authors:** Benjamin N. Laubi, Gaëtan Glauser, Erik P. Willems, Carel P. van Schaik, Judith M. Burkart

**Affiliations:** 1 Department of Evolutionary Anthropology, University of Zurich, Zürich, ZH Switzerland; 2 Department of Evolutionary Biology and Environmental Studies, University of Zurich, Zürich, ZH Switzerland; 3 Neuchâtel Platform of Analytical Chemistry, Faculty of Science, University of Neuchâtel, Neuchâtel, NE Switzerland

**Keywords:** Age effects, Androgens, Breeding roles, Callitrichids, Glucocorticoids, HPLC–MS/MS

## Abstract

Cooperative breeding presents social and reproductive challenges that differ from those associated with independent breeding, but its endocrine correlates are not well understood. We investigated cumulative steroid hormone profiles in common marmosets (*Callithrix jacchus*), a primate species characterized by shared caregiving and clearly defined reproductive roles between primary breeders and non-breeding helper group members. Using high-performance liquid chromatography-tandem mass spectrometry (HPLC–MS/MS) on hair samples, we simultaneously quantified six steroid hormones across 71 individuals varying in breeding role, sex, age, and group reproductive context. Glucocorticoids were highest in breeder females, declined with age, and were higher in breeders when dependent offspring were present. Testosterone showed a comparatively modest male–female difference and was positively associated with the presence of dependent offspring in breeders, whereas androstenedione closely paralleled testosterone across age, consistent with shared regulation. Ovarian steroids showed clear sex differentiation in progesterone, with markedly elevated levels in breeder females, whereas estradiol differences were smaller, emerging as a modest sex effect within breeders and an age effect in helpers. Together, these findings highlight weaker sexual dimorphism in sex hormone profiles than often reported for independently breeding primates. They also reveal hormonal signatures of stress and vigilance related to caregiving, consistent with cooperative breeding in marmosets. Our study demonstrates the value of hair for long-term hormonal assessment and underscores the utility of HPLC–MS/MS for quantifying multiple hormones with high specificity in socially complex primates.

**Supplementary Information:**

The online version contains supplementary material available at https://doi.org/10.1007/s10764-026-00568-9.

## Introduction

In species with independent breeding, sex differences in hormone levels often reflect distinct reproductive strategies, such as male-male competition and female investment in offspring (Wingfield *et al*., 1990). In cooperatively breeding species, by contrast, both sexes engage in caregiving and social coordination, and nonparental group members contribute to the care of others’ offspring and thus sometimes delay or even forgo reproduction (Ben Mocha *et al*., 2023; Schiel & Souto, 2017; Tardif, 1996). These differences in development and parenting roles may be accompanied by reduced or altered sex-linked hormonal differentiation relative to independently breeding species. Hormones of the hypothalamic–pituitary–adrenal (HPA) and hypothalamic-pituitary–gonadal (HPG) axes are likely to be particularly informative in this context, because glucocorticoids, androgens, estrogens, and progestogens are all involved in stress regulation, metabolism, sexual development, reproductive activity, and parental behavior (Nelson & Kriegsfeld, 2022). Examining these hormone systems may help to clarify how endocrine mechanisms support variation in social and reproductive roles in cooperative breeders.

Glucocorticoid concentrations in many group-living primates often covary with social rank; subordinates frequently have higher levels under stable dominance hierarchies (Sapolsky, 2004). In cooperative breeders, however, social differentiation is structured less by overt dominance and more by reproductive roles within the group (Schaffner & Caine, 2000). We therefore use the terminology of *breeders* and *helpers* rather than dominant and subordinate individuals to describe this distinction. In callitrichids, the only nonhuman primate family known to exhibit cooperative breeding, breeders may exhibit equal or higher baseline glucocorticoid concentrations than helpers, potentially reflecting reproductive and caregiving demands rather than subordinate stress alone (Abbott *et al*., 2003; Creel, 2001; Saltzman *et al*., 1998).

Sex differences represent another potential source of glucocorticoid variation. Among callitrichids, studies of common marmosets (*Callithrix jacchus*) have yielded inconsistent findings. Some have reported higher levels in females (Lopez *et al*., 2024; Sousa *et al*., 2015), whereas others have reported no sex differences (Phillips *et al*., 2018). Glucocorticoid profiles may also vary across age. In several primates, including callitrichids, such as common marmosets, glucocorticoid concentrations in plasma and hair have been reported to decline across adulthood, although age trajectories vary across species (Dettmer *et al*., 2014; Fourie & Bernstein, 2011; Garber *et al*., 2020; Lopez *et al*., 2024; Pryce *et al*., 2002). The presence of dependent offspring may also influence glucocorticoid levels by increasing group energetic load or social demands. However, in mammalian species with biparental care, glucocorticoid levels in males often do not differ consistently between fathers and nonbreeding males (Saltzman & Ziegler, 2014), whereas female patterns across reproductive states are more mixed (Garber *et al*., 2020; Saltzman & Maestripieri, 2011). Broader group-level effects of dependent offspring nevertheless remain understudied.

Testosterone, the best-studied androgen in primates, is often elevated in males facing strong reproductive competition, particularly in species with pronounced dominance hierarchies (Muller, 2017). In callitrichids, however, reproductive roles are shaped by cooperative breeding and overt male dominance is typically weak, which may reduce breeder-helper contrasts in testosterone. Consistent with this, adult male breeders and helpers did not differ significantly in urinary testosterone in white-faced marmosets (*Callithrix geoffroyi*) (Birnie *et al*., 2011). As in other mammals, androgen concentrations in primates are generally higher in males than in females, particularly after sexual maturity (Rege *et al*., 2019), although androgens are also present and biologically active in females, where they have received comparatively less attention (Davis & Wahlin-Jacobsen, 2015). Testosterone levels in primates typically rise during development and peak in early adulthood before declining with age (Beehner *et al*., 2009; Ellison *et al*., 2002; Sorwell & Urbanski, 2013). In male common marmosets, peak serum testosterone levels have been reported around mid-adulthood, followed by a gradual decline (Tardif *et al*., 2008).

The presence of dependent offspring may also influence testosterone, particularly in males involved in infant care (Gettler *et al*., 2011). In callitrichids, testosterone in breeding males has been reported to decrease following the birth of offspring, consistent with endocrine shifts associated with paternal responsiveness (Nunes *et al*., 2000, 2001; Ziegler *et al*., 2004, 2009), although findings are not uniform across studies (Dixson & George, 1982). Androstenedione, a weaker androgen and key precursor to both testosterone and estrogens, remains understudied in nonhuman primates (Badawy *et al*., 2021). Limited comparative data mean that expectations for androstenedione are less specific than for testosterone, but its role within the HPG axis suggests that it may provide complementary information on androgen-related endocrine variation.

In callitrichids, helper females in captivity have often been described as anovulatory, particularly in groups of unrelated co-housed individuals, which would predict lower and less variable estradiol and progesterone concentrations in helpers than breeders (Abbott & Hearn, 1978). However, evidence from natal-group and wild settings indicates that reproductive inhibition is not uniform; subordinate females may show ovarian cycling depending on age and social conditions, suggesting that endocrine differences between breeders and helpers can be context-dependent rather than absolute (Sousa *et al*., 2005; Ziegler & Sousa, 2002). Progesterone in particular may track reproductive state more strongly than social role, as suggested by recent work in black-tufted-ear marmosets (*Callithrix penicillata*) (Silva *et al*., 2024). Whether estradiol and progesterone also vary systematically between breeder and helper males remains unclear. Sex differences in estradiol and progesterone are generally expected. Females typically exhibit higher and more dynamic profiles because of ovarian cycling and broader physiological roles. However, both hormones also occur in males and can be physiologically relevant, partly through peripheral conversion pathways (Oettel & Mukhopadhyay, 2004; Vermeulen *et al*., 2002). Male marmosets can show high estradiol concentrations, and experimental evidence indicates that male estradiol reflects testicular HPG-axis activity (Nunes *et al*., 2002). Direct comparisons across sexes within the same study framework in callitrichids nevertheless remain scarce.

Age-related variation in estradiol and progesterone can reflect both pubertal maturation and reproductive aging, but patterns are not well characterized in callitrichids, particularly in males. Available evidence suggests that female marmosets can maintain ovarian steroid production into advanced age, with a late-life reproductive decline occurring relatively close to the end of lifespan (Tardif *et al*., 2008). For males, it remains unclear whether either hormone shows systematic age-related trends. The presence of dependent offspring may also be associated with variation in ovarian steroids through changes in caregiving context. Reported links between estradiol and caregiving are variable in both females and males. In callitrichids, associations with maternal behavior may be positive, negative, or absent (Fite & French, 2000; Pryce, 1993). In males, some evidence suggests that estradiol may change following offspring birth, potentially reflecting shifts in paternal behavior, although effects appear inconsistent across species and studies (Cavanaugh & French, 2013; Nunes *et al*., 2000).

Examining how steroid hormone profiles vary with these social and life-history variables requires a matrix that captures longer-term endocrine variation rather than short-term fluctuations. Matrices, such as urine and feces, can limit inference about stable baseline hormone levels, because they are influenced by circadian fluctuations and sampling constraints and because they often reflect hormone metabolites rather than circulating steroids. Hair, by contrast, reflects unconjugated hormone deposition over weeks to months and can serve as a time-integrated index of endocrine output across the period of hair growth (Behringer & Deschner, 2017). Hair has increasingly been used in primates and other mammals to quantify endocrine profiles shaped by stable demographic and social conditions (Carlitz *et al*., 2014, 2019; Grafinger *et al*., 2024; Koren *et al*., 2018). In cooperative breeding systems, where reproductive and caregiving contexts can change rapidly, this matrix may be particularly useful for characterizing longer-term endocrine profiles without requiring intensive repeated sampling.

Against this background, common marmosets provide a useful case study, because their groups typically include a primary breeding pair and multiple nonbreeding helpers, creating clear and recurring variation in reproductive role, age, and caregiving demands (Abbott *et al*., 2003; Saltzman *et al*., 2008; Sousa *et al*., 2005). In addition, their well-established use in captive research facilitates repeated sampling under known social conditions (Inoue *et al*., 2023). We therefore aimed to examine how long-term steroid hormone profiles in common marmosets vary with breeding role, sex, age, and group reproductive context (presence or absence of dependent offspring in the group). We used a high-specificity HPLC–MS/MS protocol to quantify six steroids across the HPA and HPG axes in hair samples from nonpregnant individuals, because this approach enables simultaneous multisteroid quantification with high analytical specificity and reduces problems of cross-reactivity inherent to immunoassays (Shackleton, 2010). Because comparative evidence is uneven across hormones and our analyses relied on model selection, we treated the study as a theory-guided exploratory examination of how endocrine variation is associated with cooperative breeding, while deriving directional predictions where theory provided a clear basis.

For glucocorticoids, breeder roles may entail greater reproductive and caregiving demands than helper roles and thus be associated with higher baseline HPA-axis activity. At the same time, because both sexes participate in caregiving and group coordination, major sex differences are not necessarily expected. Accordingly, we predicted higher cortisol and corticosterone concentrations in breeders than in helpers and no consistent sex differences. We also examined age-related variation, including a possible decline across adulthood, but did not make a directional prediction for the effect of dependent-offspring presence. Because cortisol is generally considered the predominant glucocorticoid in primates, we further predicted corticosterone to occur at lower concentrations and to show broadly similar directional patterns while potentially varying partly independently of cortisol (Koren *et al*., 2012; Novak *et al*., 2013).

For androgens, the weak dominance hierarchies and shared caregiving characteristic of callitrichid cooperative breeding led us to predict only modest breeder-helper differences in testosterone; breeders tended to show slightly higher concentrations than helpers. Sex differences should nonetheless persist because of gonadal differences in androgen production. We therefore still predicted higher testosterone concentrations in males than in females. We also predicted developmental increases with age followed by possible later decline, reflecting maturation and subsequent adult change, and lower testosterone concentrations in the presence of dependent offspring, particularly in caregiving males, if paternal demands suppress androgen output. Because comparative data on androstenedione are particularly limited, we did not make strong directional predictions for this hormone.

For estradiol and progesterone, differentiated reproductive roles led us to predict divergence in ovarian steroid profiles between breeders and helpers, with higher concentrations in female breeders than in female helpers, although this contrast was expected to be weaker and more variable in our sample, because helpers were related natal-group members and reproductive inhibition can be incomplete in such settings. Because ovarian cycling should also produce higher and more variable concentrations in females than in males overall, we further predicted higher concentrations in females than in males. As gonad-dependent hormones, estradiol and progesterone were also expected to increase with maturation, with the possibility of late-life declines in females reflecting reproductive aging, while we did not make a corresponding prediction of age-related declines in males. Because evidence linking ovarian steroids to caregiving context is mixed, we did not make a strong directional prediction for dependent-offspring presence.

## Methods

### Samples, Animals, and Housing

We analyzed 193 hair samples of 71 common marmosets, including 40 females and 31 males (Supplementary Table 1). Helpers were related group members, typically offspring of the breeding female. We did not collect samples from pregnant females. In this colony, we prevented pregnancy in breeding females by administering cloprostenol (a prostaglandin F2α analogue; 0.5 µg) intramuscularly at approximately 6-week intervals to induce luteolysis. Thus, females that we classified as nonpregnant remained endocrinologically active, with ovarian function maintained and cycle timing periodically reset by cloprostenol treatment under this management regime. We collected eleven of the 193 samples from individuals living in groups with dependent offspring (< 6 months). To ensure that hormone levels reflected stable social and reproductive states, we only included samples from animals whose breeding role and group composition remained unchanged during the interval between the previous shave and collection of the regrown hair sample. At the time of sampling, the age of the individuals ranged from 215 days to 19.5 years (median = 5.46 years), and they lived in 22 groups of two to seven individuals.

We housed all animals in heated indoor enclosures with ad libitum access to outdoor enclosures on days when temperatures exceeded 15 °C. In addition to the natural lighting, indoor housing included artificial lighting and UV lamps (300 W) on a 12 h/12 h light–dark cycle. We enriched both indoor and outdoor enclosures with natural branches, climbing structures, wood chips, or soil with natural vegetation. The diet included vitamin-enriched rice porridge in the morning, vegetables at midday, and a protein-rich afternoon snack consisting of animal protein (boiled egg, fish, or chicken) or crystallized gum arabic. We kept the feeding regimen consistent across all groups throughout the study period: we standardized breakfast, while we varied the vegetables and the protein snack within a predefined set of items as part of routine husbandry, without systematic between-group differences or sustained temporal changes.

### Sampling Procedure

We collected hair samples during regular shavings that we performed for visual individual recognition. We shaved an area of about 3 cm × 3 cm just above the tail base on the lower back using a battery-operated veterinarian trimmer (Aesculap, Isis). We trimmed the whole area evenly so that we would sample only freshly regrown hair at the next collection. We then placed the hair samples into small envelopes of baking paper inside a zip-lock bag and stored the bag in an opaque paper envelope together with information on the individual, group, and date of collection. We kept the envelopes dry and at room temperature until analysis. Because hair growth rates varied substantially between individuals, we could not apply a fixed sampling interval (Supplementary Table 1). Instead, we adjusted the timing individually and collected new samples once the shaved area had almost fully regrown (approximately 3 cm) but was still visually distinguishable from the surrounding fur. This approach resulted in a mean growth duration of 7.61 months (SD = 3.61 months) across individuals.

### Sample Preparation and Steroid Extraction

We first washed hair samples with 2 ml of isopropanol for 20 s (Davenport *et al*., 2006), centrifuged them (up to 12,000 rpm), discarded the solvent, and then dried them in a SpeedVac (40 °C for ~ 30 min). We chose this procedure based on preliminary internal testing on a small subset of samples, which confirmed minimal hormone loss beyond the first wash (Supplementary Tables 2–4). For steroid extraction, we adapted and optimized the protocol developed by Glauser and Vuilloud (Vullioud, 2018) at the Neuchâtel Platform of Analytical Chemistry (NPAC) for our samples. After washing and drying, we added three 3.5 mm of UFO steel beads to each Eppendorf tube containing 10 mg (± 0.3 mg) of washed hair. We then ground the samples for 4 min at 27 Hz with a 400 MM Qiagen TissueLyser II bead mill. After a break of 1 min, we ground the samples for an additional 4 min. We then centrifuged the tubes for 2 min at 15,000 rpm to ensure the powder fell to the bottom, performed a third round of grinding, and centrifuged the tubes again for 2 min at 15,000 rpm. We added 1 ml of methanol (MeOH) containing the internal standards (0.5 ng/ml for all hormones, except testosterone at 0.2 ng/ml) to the pulverized samples and shook the tubes for 4 min at 27 Hz with the tissue lyser for extraction. After centrifuging the samples for 2 min at 15,000 rpm, we transferred the supernatant to a new 2-ml Eppendorf tube. We repeated the extraction step with 0.8 ml MeOH (not containing any internal standard), combined the supernatants and dried them down in the SpeedVac (40 °C for about 60 min). We reconstituted the dried extracts in 1 ml 5% MeOH before further purification using solid-phase extraction. For this step, we conditioned a Discovery DSC-18 cartridge (100 mg) with 1 ml MeOH, equilibrated it with 1 ml of 5% MeOH, loaded the sample, washed the cartridge successively with 1 ml of 5% MeOH and 1 ml of hexane and eluted it with 1 ml of ethyl acetate. We further purified the ethyl acetate fraction using a liquid–liquid extraction with 0.1% NH₄OH. We then dried down the organic phase again (40 °C for about 45 min) and subsequently resuspended it in 100 μl of 50% MeOH. After centrifuging the samples for 3 min centrifugation at 13,000 rpm, we transferred 70 μl of the supernatants to HPLC vials.

### High-performance Liquid Chromatography Mass Spectrometry (HPLC – MS/MS)

We quantified steroids using an Acquity UPLC™ I-Class system coupled to a Xevo TQ-XS triple quadrupole mass spectrometer (Waters, Milford, MA) through an electrospray interface in positive ionization mode. We controlled the system by MassLynx 4.2 (Waters). We separated steroids on an Acquity UPLC BEH C18 column (50 × 2.1 mm i.d., 1.7-μm particle size, Waters) at a flow rate of 0.4 ml/min and at 30 °C. The mobile phases were milli-Q water and formic acid 0.05% (mobile phase A) and acetonitrile and formic acid 0.05% (mobile phase B). We applied the following gradient program: 10–56.2% B in 7.5 min, 56.2–100% B in 0.1 min, 100% B for 1 min, and 10% B for 1.5 min. We diverted the HPLC flow from the mass spectrometer into the waste from 0.0–3.28 min, then from 6.03–7.08 min, and from 7.73 to the end of the run. The injection volume was 2.5 µl. The source mass spectrometer parameters were as follows: capillary voltage + 1.5 kV, desolvation temperature 600 °C, cone gas flow 350 l/hr, desolvation gas flow 1000 l/hr. We optimized MRM transitions, cone, and collision energies for each steroid (Supplementary Table 5). We prepared calibration solutions in 50% methanol at concentrations of 0.02, 0.2, 1, 4, 20, 50, and 100 ng/mL for corticosterone, testosterone, androstenedione, and 11-ketotestosterone, and at 0.1, 1, 5, 20, 100, 250, and 500 ng/ml for cortisol, cortisone, estradiol, and progesterone, along with internal standards.

We quantified steroids by integrating chromatographic peaks using TargetLynx™ software, with normalization to the internal standards. To ensure accuracy, we manually reviewed peak integration for each hormone and sample. We generated calibration curves for each batch of samples, applying the most suitable model (linear or quadratic) and weighting factor (typically 1/x) depending on the calibration data. All measured concentrations were above the method's limit of quantification, which we determined based on a signal-to-noise ratio of 10.

### Method Validation

We evaluated method performance using a charcoal-stripped pooled hair matrix, because steroid-free (“blank”) marmoset hair is not available and endogenous steroid concentrations in unmanipulated hair are too high for background-corrected spiking (Voegel *et al*., 2021). For matrix preparation, we processed hair from 12 individuals equally spanning sexes and breeding roles (Sample IDs: 85, 99, 107, 112, 115, 119, 123, 179, 189, 221, 277, 292; Supplementary Table 1) using the same washing, homogenization, extraction, and clean-up procedures that we described above for study samples. For each individual, we performed two independent extractions. We then treated extracts individually with 10 mg (± 1 mg) activated charcoal per extract to strip endogenous steroids, filtered them to remove charcoal, and combined them into a pooled stripped matrix only after charcoal removal. We aliquoted the pooled stripped matrix and spiked it at three different concentrations for each steroid (Supplementary Table 6), after which we processed the samples through the same downstream SPE and liquid–liquid extraction steps as study samples and analyzed them to estimate LOD/LOQ, accuracy, and precision. We defined LOD and LOQ concentrations which gave signal-to-noise ratios in the samples of three and ten, respectively. When we found no sample at such levels in the dataset, we extrapolated by cross multiplication. We determined accuracy (n = 5) by calculating the percentage of deviation between expected and measured concentrations at the three concentration levels. We calculated the percentage of relative standard deviation as the measure of precision (n = 5). Charcoal stripping effectively removed endogenous steroids in a pilot test. However, post-spike recovery losses for some analytes were consistent with carryover of residual active charcoal in the stripped pool, which may have continued to adsorb steroids after spiking.

We initially targeted eight steroids. Six met the prespecified acceptance criteria (accuracy 80–120% across QC levels). Cortisone and 11-ketotestosterone showed persistently low post-spike recoveries in the charcoal-stripped matrix and we report them only in the supplementary materials (Supplementary Figs. 9b, 10c, 11c, 16; Supplementary Tables 10, 11, 12, 20, 21, 22). For the complete validation summary, see Supplementary Table 6.

### Data Analysis

We conducted statistical analyses by using R version 4.3.3 (R Core Team, 2024). To account for the age dependence of breeding status, where only mature individuals can be breeders and no helpers exceed 9 years of age in our dataset, we restricted the analysis to the age range shared by helpers and breeders. This shared age-range dataset formed the basis of our main analysis (N = 101 samples out of 193 total from 44 individuals; Table I). Additionally, we performed separate analyses on subsets restricted to helpers (N = 89 samples from 36 individuals) and breeders (N = 104 samples from 37 individuals). Individuals may contribute to more than one analysis subset. This approach enabled a detailed investigation of age-related patterns specific to each breeding role. For each dataset, we fitted linear mixed-effects models using the ‘lmer’ function of the lme4 package. For each hormone, we modeled the logarithm of its *concentration* (log1p-transformed to manage zero values) as a function of the following predictors: *role* (breeder or helper), *sex* (male or female), *age* (in days, and standardized to z-scores to aid model convergence), *presence of dependent offspring* (True vs. False), and the *batch number* of hormonal extraction (1 or 2). In the helper-only subset, we excluded the offspring variable because it contained only one case. To capture potential nonlinear effects of age, we applied a natural spline with four degrees of freedom to androgens, estradiol, and progesterone in the helper and breeder subsets. In the shared age-range model, which we restricted to the age range shared by breeders and helpers, we modeled age linearly because of the lower resolution. We included crossed random effects for *group identity* and *individual identity* to account for repeated measures and data hierarchy. To test for interactive effects, we initially included a three-way interaction among *role*, *sex*, and *age* for each hormone. Since the data did not support these full models, we used Akaike’s Information Criterion (AIC) to guide the selection of simplified exploratory models, retaining all main effects but reducing interaction complexity as needed. We also considered hair growth duration (days since the last shave) as a covariate, but excluded it from the final models because it neither improved model fit nor changed inference for the focal predictors.

**Table I:** Sample composition and age distribution across analysis subsets in common marmosets from a captive colony at the University of Zurich, Switzerland, sampled between November 2021 and December 2023

Category	Samples (n)	Individuals (n)	Samples/ind. (median [min–max])	Age median [IQR] (y)	Age range (y)
Shared age-range dataset					
Female breeders	28	10	2 [1–5]	6.8 [5.2–7.7]	2.4–8.5
Male breeders	29	10	3 [1–5]	5.5 [4.2–6.7]	3.1–8.7
Female helpers	29	16	2 [1–4]	4.1 [3.3–6.1]	2.4–8.7
Male helpers	15	8	2 [1–3]	5.4 [2.7–6.1]	2.4–7.3
Total	101	44	2 [1–5]	5.4 [3.7–6.9]	2.4–8.7
Breeders-only dataset					
Female breeders	50	18	2 [1–6]	8.3 [6.7–10.5]	2.4–20.8
Male breeders	54	19	2 [1–5]	8.2 [5.2–10.5]	3.1–19.9
Total	104	37	2 [1–6]	8.3 [5.6–10.6]	2.4–20.8
Helpers-only dataset					
Female helpers	53	23	2 [1–5]	2.7 [1.5–4.3]	0.6–8.7
Male helpers	36	13	3 [1–4]	2.3 [1.7–3.4]	0.7–7.3
Total	89	36	2 [1–5]	2.4 [1.7–4.1]	0.6–8.7

The shared age-range dataset comprised individuals aged 870 to 3183 days (inclusive). “Samples/individual” indicates repeated sampling within each subset. Individuals could contribute to more than one subset. We present ages in years for readability; for statistical analyses we used age in days. Full sample details are provided in Supplementary Table 1

### Use of Large Language Models

This manuscript benefited from the use of ChatGPT-4 (OpenAI, 2024) to improve clarity, grammar, and readability. After using this tool/service, the authors reviewed and edited the content as needed and take full responsibility for the content of the publication.

## Ethical Note

All procedures involving animals were conducted in accordance with Swiss animal welfare regulations and were approved by the Cantonal Veterinary Office of Zurich (license number 35517). The authors declare that they have no conflict of interest.

### Data Availability

The dataset generated and analyzed in this study is available in the Open Science Framework (OSF) repository at https://doi.org/10.17605/OSF.IO/R6AQB.

## Results

Unless stated otherwise, results refer to the shared age-range model fitted to the dataset restricted to the age range shared by breeders and helpers (N = 101 samples from 44 individuals). We fitted role-specific models to the helper-only (N = 89 samples from 36 individuals) and breeder-only (N = 104 samples from 37 individuals) subsets (Table I); Supplementary Tables 7 to 28 provide the full model outputs.

### Glucocorticoids

For both glucocorticoids, we selected final models based on Akaike’s Information Criterion (AIC); these models included main effects and selected two-way interactions. These exploratory models performed significantly better than the corresponding null-models, which contained fixed and random intercepts only (Cortisol: χ^2^ = 63.522, df = 6, *p* < 0.001, R^2^_c_ = 0.744; Corticosterone: χ^2^ = 66.92, df = 6, *p* < 0.001, R^2^_c_ = 0.796). The same was true for the models of the two sub-datasets that included exclusively breeders or helpers (Supplementary Tables 7–9; 13–15).

**Fig. 1:**
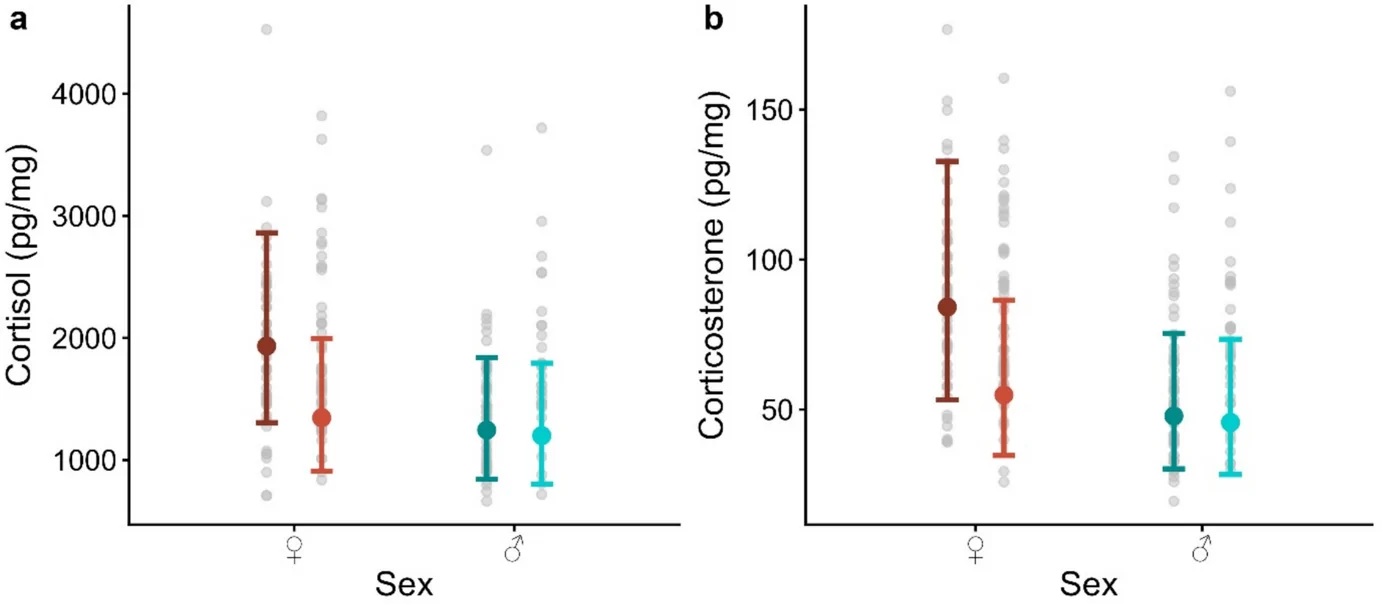
Hair cortisol (**a**) and corticosterone (**b**) concentrations in common marmosets from a captive colony at the University of Zurich, Switzerland, sampled between November 2021 and December 2023, shown for the shared age range of breeders and helpers. Gray points show raw values. Colored points show model-predicted values, and error bars show 95% confidence intervals. Colors indicate female breeders (brown), female helpers (red), male breeders (blue), and male helpers (turquoise). Likelihood-ratio test statistics for the sex-by-breeding-role interaction were cortisol, χ^2^LRT(1) = 12.8, *p* < 0.001; and corticosterone, χ^2^LRT(1) = 7.28, *p* < 0.01. The two panels use different y-axis scales.

For cortisol and corticosterone, we detected significant interactions between breeding role and sex (Fig. 1; Cortisol: χ^2^ = 12.8, df = 1, *p* < 0.001; Corticosterone: χ^2^ = 7.281, df = 1, *p* < 0.01), indicating that female breeders exhibited higher concentrations than male breeders and helpers of either sex. Role-stratified analyses showed that the cortisol sex difference was driven primarily by breeders (Supplementary Fig. 9), with no significant sex effect among helpers. In contrast, corticosterone followed a comparable pattern in both breeders and helpers, with additional age-related structure in breeders.

**Fig. 2:**
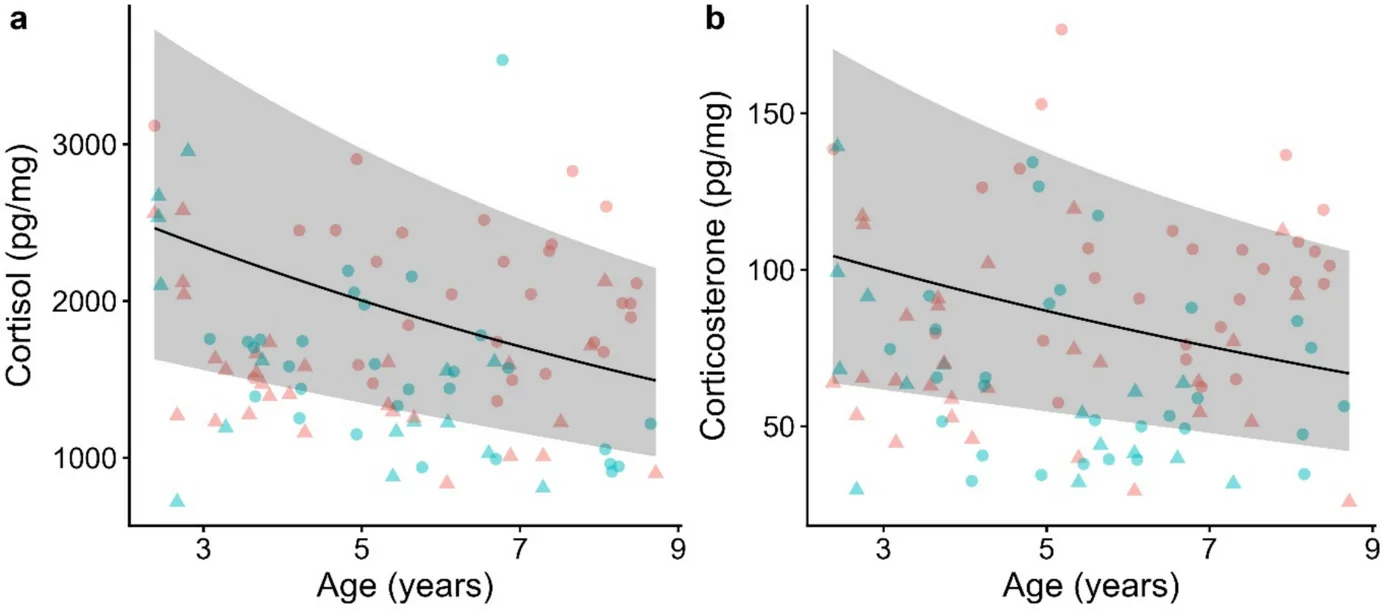
Hair cortisol (**a**) and corticosterone (**b**) concentrations in common marmosets from a captive colony at the University of Zurich, Switzerland, sampled between November 2021 and December 2023, shown for the shared age range of breeders and helpers. Points show raw values. Black lines show model-predicted values, and shaded areas show 95% confidence intervals. Colors indicate sex (red = females, turquoise = males), and symbols indicate breeding role (circles = breeders, triangles = helpers). Likelihood-ratio test statistics for the age effect were cortisol, χ^2^LRT(1) = 26.43, *p* < 0.001; and corticosterone, χ^2^LRT(1) = 10.4, *p* < 0.01. The two panels use different y-axis scales.

In the shared age-range dataset, both cortisol and corticosterone concentrations decreased significantly with age (Fig. 2; Cortisol: χ^2^ = 26.433, df = 1, *p* < 0.001; Corticosterone: χ^2^ = 10.399, df = 1, *p* < 0.01). This age-related decline was also evident in the breeders-only and helpers-only datasets (Supplementary Figs. 10 and 11), with one qualification: in breeders, the corticosterone decrease occurred in females only. Male breeders showed consistently low corticosterone levels across age, whereas female breeders started at higher levels and declined with age toward male-like concentrations (Supplementary Fig. 10b).

**Fig. 3:**
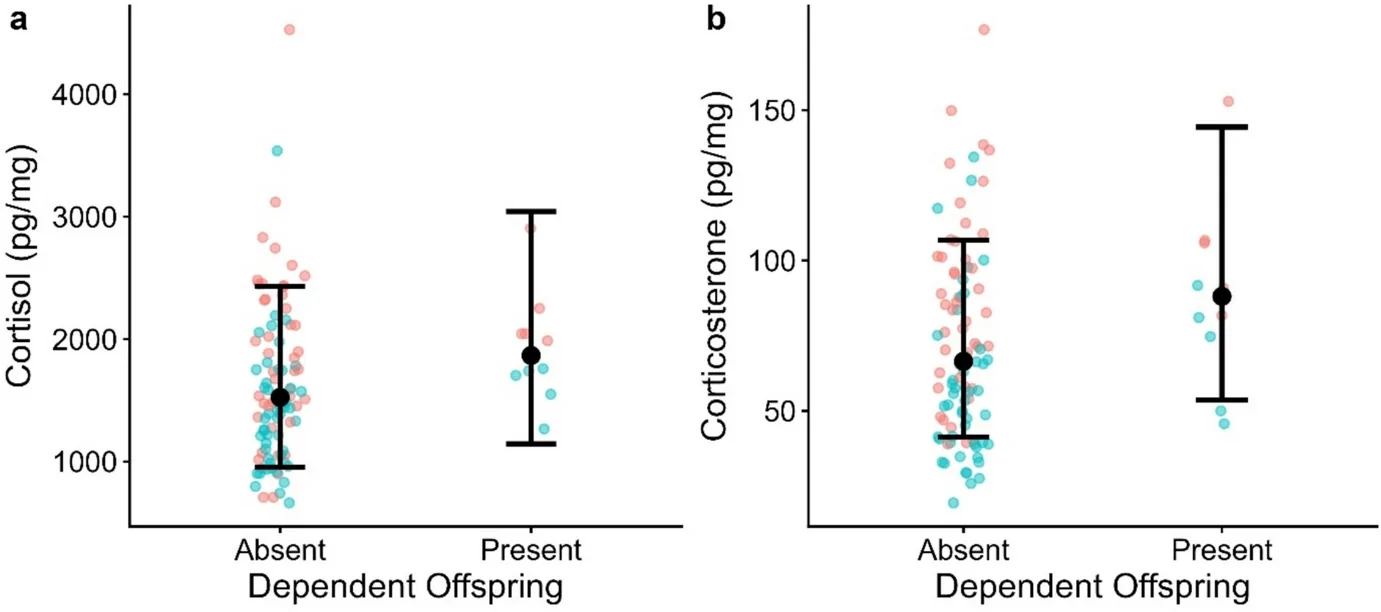
Hair cortisol (**a**) and corticosterone (**b**) concentrations in common marmosets from a captive colony at the University of Zurich, Switzerland, sampled between November 2021 and December 2023, shown for breeders only. Colored points show raw values. Black points show model-predicted values, and error bars show 95% confidence intervals. Colors sex (red = females, turquoise = males). Likelihood-ratio test statistics for the effect of dependent offspring presence in the group were cortisol, χ^2^LRT(1) = 5.5, *p* < 0.05; and corticosterone, χ^2^LRT(1) = 10.33, *p* < 0.01. The two panels use different y-axis scales.

Finally, we detected significant main effects of the presence of dependent offspring on glucocorticoid levels in the breeder subsets (Fig. 3; Cortisol: χ^2^ = 5.502, df = 1, *p* < 0.05; Corticosterone: χ^2^ = 10.33, df = 1, *p* < 0.01). These findings show that the presence of dependent offspring is associated with elevated levels of both glucocorticoids in breeding individuals. In corticosterone, this effect was strong enough to also appear in the shared age-range dataset, which included breeders and helpers (Supplementary Fig. 12).

### Androgens

For testosterone, the final model including only the main effects significantly outperformed the null model with the same random structure but containing no fixed effects (χ^2^ = 14.143, df = 5, *p* < 0.05, R^2^c = 0.586). However, for androstenedione, no model that we fitted to either the shared age-range dataset (χ^2^ = 10.77, df = 5, *p* = 0.056, R^2^c = 0.656) or the breeders-only dataset (χ^2^ = 11.50, df = 7, p=0.118, ${R}_{c}^{2}=0.376$) outperformed the null model. Consequently, we limited our analysis to the helper dataset, where the fitted model significantly outperformed the null model (χ^2^ = 58.73, df = 6, *p* < 0.001, R^2^c = 0.763).

**Fig. 4:**
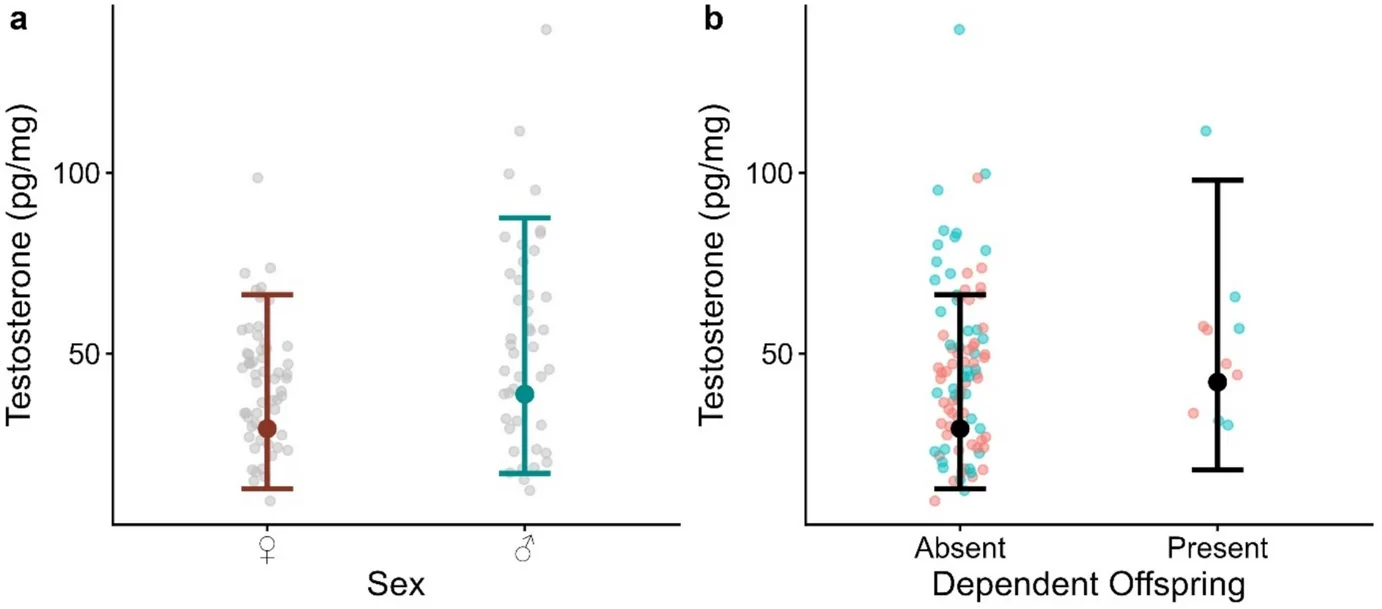
Hair testosterone concentrations in common marmosets from a captive colony at the University of Zurich, Switzerland, sampled between November 2021 and December 2023, shown for the shared age range of breeders and helpers. **a** Testosterone concentrations by sex. Gray points show raw values, colored points show model-predicted values, and error bars show 95% confidence intervals; colors indicate sex (brown = females, blue = males). **b** Testosterone concentrations by dependent-offspring presence in the group. Colored points show raw values, black points show model-predicted values, and error bars show 95% confidence intervals; colors indicate sex (red = females, turquoise = males). Likelihood-ratio test statistics were sex, χ^2^LRT(1) = 5.18, *p* < 0.05; and dependent offspring presence, χ^2^LRT(1) = 5.71, *p* < 0.05.

**Fig. 5:**
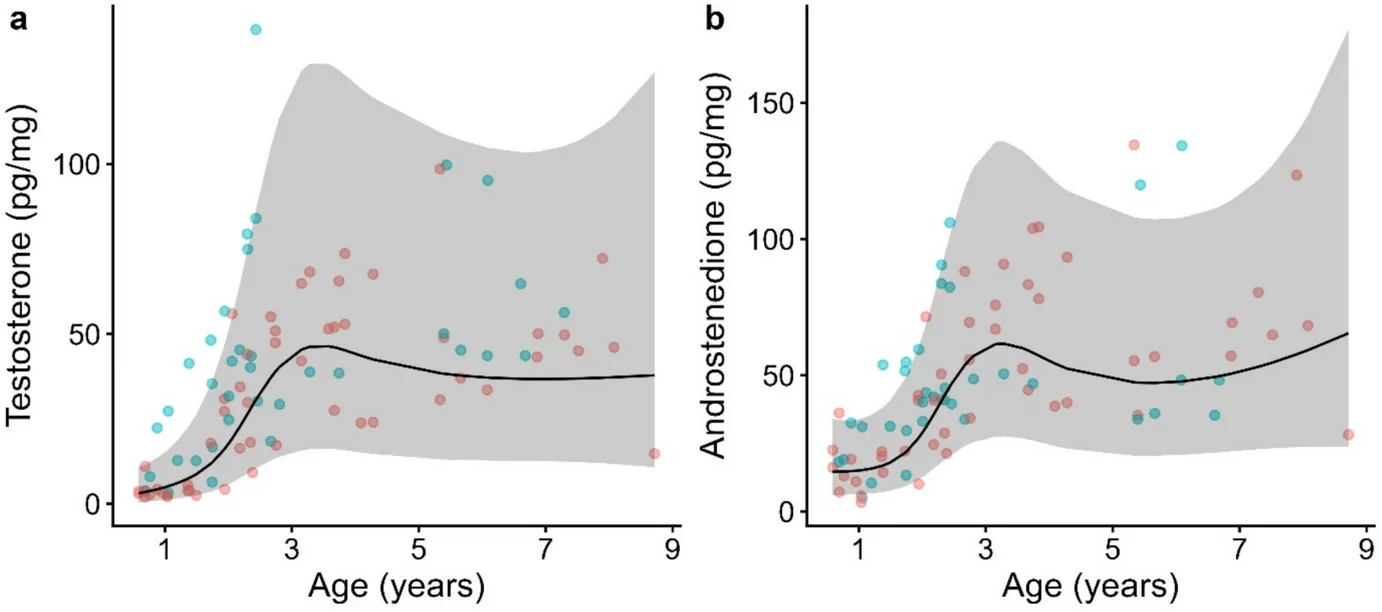
Hair testosterone (**a**) and androstenedione (**b**) concentrations in common marmosets from a captive colony at the University of Zurich, Switzerland, sampled between November 2021 and December 2023, shown for helpers only. Points show raw values. Black lines show model-predicted values, and shaded areas show 95% confidence intervals. Colors indicate sex (red = females, turquoise = males). Likelihood-ratio test statistics for the age effect were testosterone, χ^2^LRT(4) = 162.87, *p* < 0.001; and androstenedione, χ^2^LRT(4) = 68.66, *p* < 0.001.

In the shared age-range dataset, testosterone concentrations were significantly higher in males than in females (Fig. 4a; χ^2^ = 5.182, df = 1, *p* < 0.05). This pattern was consistent in both the breeder-only and helper-only datasets (Supplementary Fig. 13). Testosterone concentrations were also higher when dependent offspring were present (Fig. 4b; χ^2^ = 5.705, df = 1, *p* < 0.05), and the same association appeared in the breeder-only dataset (Supplementary Fig. 14). When restricting the analysis to helpers, we additionally detected a significant effect of age (Fig. 5a; χ^2^ = 162.868, df = 4, *p* < 0.001), with testosterone levels increasing over approximately the first three years of life before reaching a plateau.

We limited the androstenedione analysis to the helper-only dataset because of model constraints. The model revealed a significant age effect (Fig. 5b; χ^2^ = 68.656, df = 4, *p* < 0.001), similar to the pattern found in testosterone among helpers. Concentrations initially rose, reaching a peak at around 3 years of age. After this peak, levels generally stabilized, with minor fluctuations, maintaining a relatively consistent level through to approximately 9 years.

### Estradiol ad Progesterone

Exploratory models for estradiol and progesterone both performed significantly better than the corresponding null-models containing only random effects (estradiol: χ^2^ = 13.062, df = 5, *p* < 0.05, R^2^_c_ = 0.317; progesterone: χ^2^ = 78.319, df = 6, *p* < 0.001, R^2^_c_ = 0.953).

**Fig. 6:**
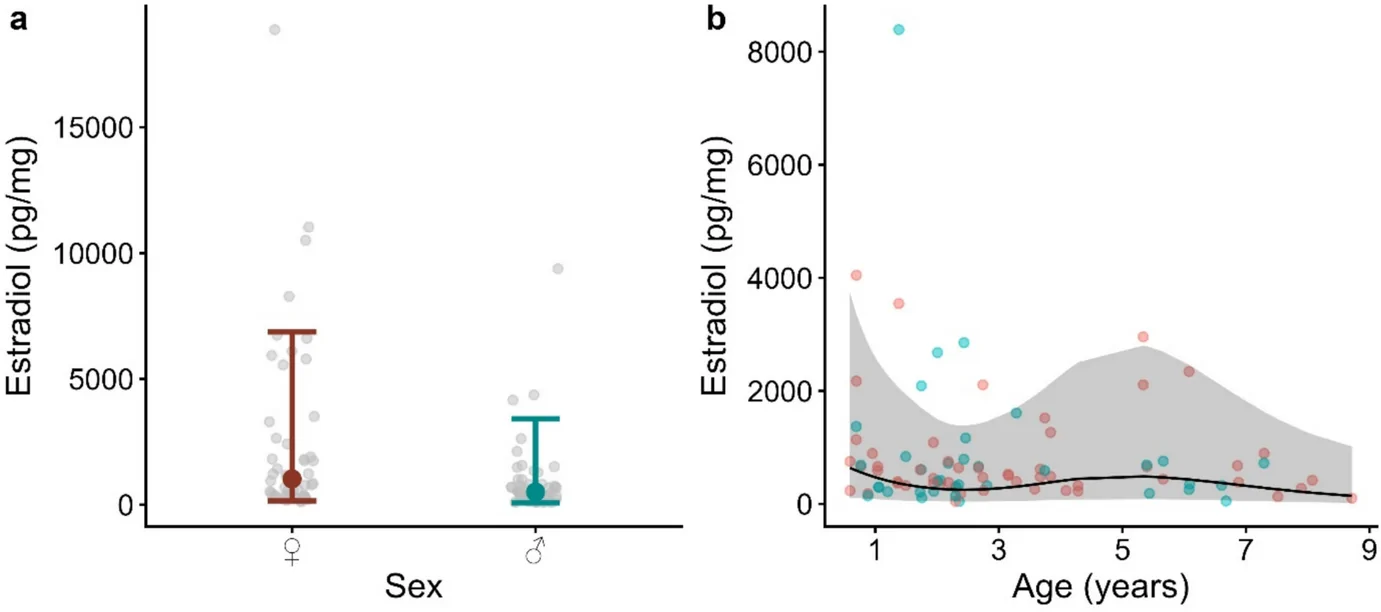
Hair estradiol concentrations in common marmosets from a captive colony at the University of Zurich, Switzerland, sampled between November 2021 and December 2023. **a** Estradiol concentrations by sex in breeders only. Grey points show raw values, colored points show model-predicted values, and error bars show 95% confidence intervals. Colors indicate sex (brown = females, blue = males). **b** Estradiol concentrations across age in helpers only. Colored points show raw values. The black line show model-predicted values, and shaded areas show 95% confidence intervals. Colors indicate sex (red = females, turquoise = males). Likelihood-ratio test statistics were sex in breeders, χ^2^LRT(1) = 12.26, *p* < 0.001; and age in helpers, χ^2^LRT(4) = 9.52, *p* < 0.05. The two panels use different y-axis scales.

For estradiol, the best-supported model did not include interaction terms. In the shared age-range dataset, breeding role, sex, age, and dependent-offspring presence did not show statistically significant main effects (all *p* > 0.05; Supplementary Table 23). In the stratified analyses, however, we detected a significant sex effect in the breeder-only dataset, with female breeders showing higher estradiol levels than male breeders (Fig. 6a; χ^2^ = 12.263, df = 1, *p* < 0.001). In the helper-only dataset, the natural spline term for age was significant (Fig. 6b; χ^2^ = 9.517, df = 4, *p* < 0.05), indicating modest nonlinear age-related variation: predicted estradiol levels dipped slightly at younger ages, increased modestly through midlife, and then declined somewhat at older ages.

**Fig. 7:**
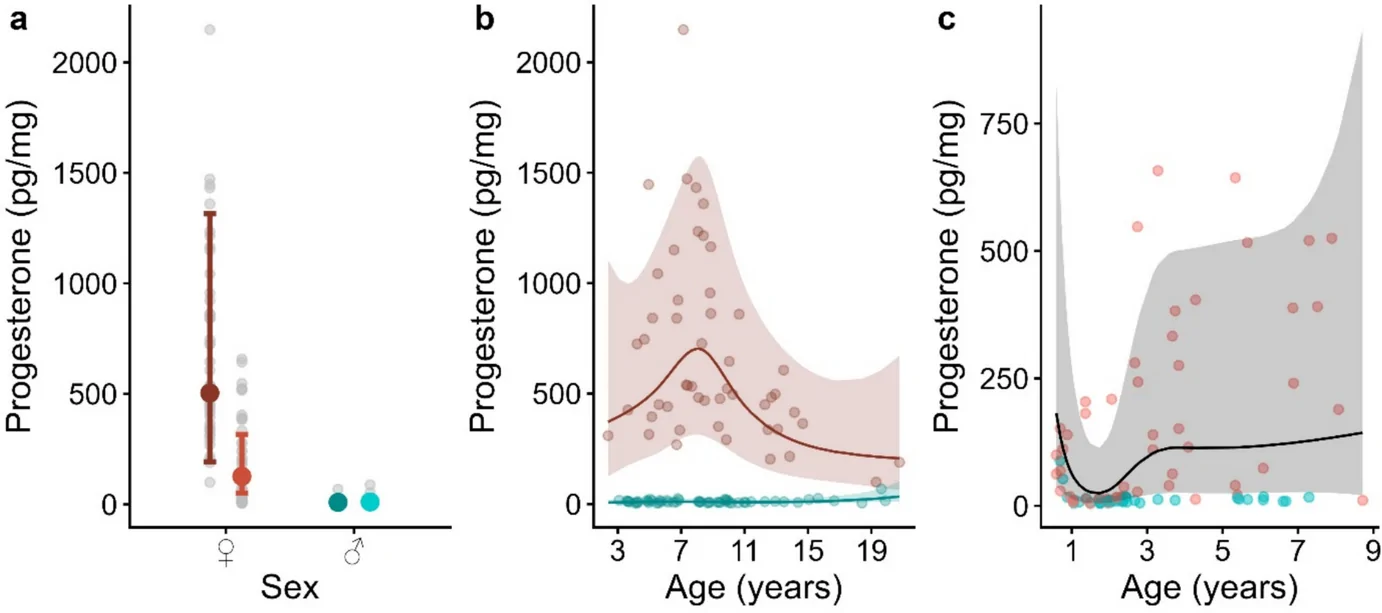
Hair progesterone concentrations in common marmosets from a captive colony at the University of Zurich, Switzerland, sampled between November 2021 and December 2023. **a** Progesterone concentrations in the shared age range of breeders and helpers. Gray points show raw values; colored points and error bars show model-predicted values and 95% confidence intervals. Colors indicate female breeders (brown), female helpers (red), male breeders (blue), and male helpers (turquoise). **b** Progesterone concentrations across age and sex in breeders only. Points show raw values. Lines and shaded areas show model-predicted values and 95% confidence intervals. Colors indicate sex (brown = females, blue = males). **c** Progesterone concentrations across age in helpers only. Points show raw values. Black lines and shaded areas show model-predicted values and 95% confidence intervals. Colors indicate sex in the raw data (red = females, turquoise = males). Likelihood-ratio test statistics were sex-by-breeding-role interaction, χ^2^LRT(1) = 9.32, *p* < 0.01; and sex-by-age interaction in breeders, χ^2^LRT(4) = 22.03, *p* < 0.001; age effect in helpers, χ^2^LRT(4) = 32.62, *p* < 0.001. Panel **c** uses a different y-axis scale from panels **a** and **b**.

For progesterone, the best-fitting model included a significant two-way interaction between sex and role (Fig. 7a; χ^2^ = 9.324, df = 1, *p* < 0.01). Overall, females showed substantially higher progesterone concentrations than males in both breeding roles. This sex difference was greatest among breeders because female breeders exhibited markedly elevated progesterone levels, whereas males remained consistently low and did not differ between breeding roles.

In the breeders-only dataset, we detected an additional significant two-way interaction between sex and age (Fig. 7b; χ^2^ = 22.025, df = 4, *p* < 0.001). This interaction revealed that progesterone levels in female breeders increase with age, peaking around 8 years and subsequently decline. In contrast, male progesterone levels remain consistently low throughout their lifespan.

In the helper-only dataset, progesterone was significantly higher in females than in males (Supplementary Fig. 15; χ^2^ = 19.014, df = 1, *p* < 0.001) and showed significant non-linear age-related variation (Fig. 7c; χ^2^ = 32.624, df = 4, *p* < 0.001). Predicted levels were relatively high early in life, declined to a trough at approximately 1.5 years, and then increased again to reach a stable plateau from around 3 years onward.

## Discussion

Long-term steroid hormone profiles in common marmosets showed the clearest associations with reproductive role and caregiving context in glucocorticoids and progesterone, whereas androgen differences were comparatively modest. Female breeders showed the highest glucocorticoid concentrations, both glucocorticoids declined with age, and breeders had higher glucocorticoid levels when dependent offspring were present. Testosterone differed only modestly between males and females and was positively associated with dependent-offspring presence, whereas testosterone and androstenedione in helpers showed similar age-related increases consistent with developmental maturation. Among ovarian steroids, progesterone showed the clearest sex- and role-linked differentiation, with markedly elevated concentrations in breeder females, whereas estradiol differences were weaker and emerged mainly as a sex effect in breeders. Both ovarian steroids also showed age-related variation within role-specific subsets.

### Glucocorticoids

Our findings both corroborate and diverge from existing research on glucocorticoid hormones in common marmosets. Female breeders displayed significantly higher hair cortisol and corticosterone concentrations than any other demographic group, aligning with some studies (Garber *et al*., 2020; Saltzman, 2004), although others have reported no sex differences in marmoset hair glucocorticoids (Phillips *et al*., 2018). These sex- and role-associated differences are unlikely to be driven solely by gestational physiology, as our analyses excluded pregnant females. They also are difficult to reconcile with the historically prominent notion of stress-mediated reproductive suppression or inhibition of helper females (Saltzman *et al*., 2008), although this should be interpreted cautiously because helpers in our study were primarily natal daughters, for whom suppression is not necessarily complete or uniform (Ziegler & Sousa, 2002). The elevated glucocorticoids in females, particularly breeders, may reflect sex differences in longer-term social and reproductive demands, consistent with field observations that female marmosets show pronounced reproductive competition while males often engage more strongly in cooperative roles (Yamamoto *et al*., 2014).

Consistent with previous work in common marmosets, we observed a clear age-associated decline in both cortisol and corticosterone concentrations (Lopez *et al*., 2024; Pryce *et al*., 2002). Similar age-related decreases in glucocorticoids have been reported in lemurs and other monkey species, such as vervet monkeys (*Chlorocebus pygerythrus*), rhesus macaques (*Macaca mulatta*), and Guinea baboons (*Papio papio*) (Dettmer *et al*., 2014; Fourie & Bernstein, 2011; Laudenslager *et al*., 2012; Tennenhouse *et al*., 2017). By contrast, studies in humans and chimpanzees (*Pan troglodytes*) more often report increasing glucocorticoid levels across adulthood, particularly in males (Cauter *et al*., 1996; Emery Thompson *et al*., 2020; Fedurek *et al*., 2020, 2023; Feller *et al*., 2014; Gust *et al*., 2000; Roelfsema *et al*., 2017). These divergent patterns may reflect differences in life-history pace and aging biology: in shorter-lived primates, reduced reproductive effort and changing social demands in later life may be accompanied by downregulation of HPA-axis activity, whereas in long-lived hominids, cumulative exposure to social and ecological challenges may contribute to increasing glucocorticoid output or dysregulation with age (Fischer, 2017; Gupta, 2019).

Moreover, contrary to previous findings based on hair samples analyzed with enzyme immunoassay (Garber *et al*., 2020), we detected a positive association between the presence of dependent offspring in the group and glucocorticoid concentrations in breeders. This discrepancy likely reflects differences in how reproductive context was operationalized. Garber *et al*. (2020) categorized females by reproductive condition (pregnant, lactating, or recently parturient), whereas we excluded pregnant females and instead modelled dependent-offspring presence at the group level, regardless of an individual’s reproductive state. Differences in analytical approach may also partly contribute, because HPLC–MS/MS provides higher chemical specificity than immunoassays and may detect more subtle variation in hair glucocorticoids. We collected only eleven samples in our dataset during periods when dependent offspring were present, limiting our ability to disentangle lactation-related physiology from broader caregiving and social demands. Accordingly, the breeder-level association may partly reflect maternal lactation in the subset of females sampled during presence of suckling infants. However, the absence of a sex-specific offspring effect within breeders suggests that offspring-related demands affect both mothers and fathers equally in this biparental care system. This pattern is consistent with the idea that dependent offspring increase sustained social and energetic demands in cooperative breeders, including heightened coordination, vigilance, and carrying effort (Schradin & Anzenberger, 2001), especially in breeders who bear the primary burden of care. Finally, the age-dependent decline in glucocorticoids suggests that these pressures may lessen as breeders age, potentially reflecting increased experience and buffering via helper support in groups with older breeders.

Although corticosterone occurred at much lower concentrations than cortisol, both hormones showed broadly parallel patterns across predictors in our dataset. Comparative work indicates that, despite strong species-level covariance, cortisol and corticosterone can be weakly correlated among individuals within a species in some taxa (Koren *et al*., 2012); our results suggest tighter coupling in common marmosets under the conditions studied here.

### Androgens

We found no evidence that breeding role differentiated androgen profiles in either sex; breeders and helpers did not differ significantly in testosterone. This pattern aligns with the prediction that in cooperative breeders, testosterone may not track reproductive role in the same way as in hierarchically structured, independently breeding primates (Muller, 2017). Consistent with our results, adult male breeders and helpers also did not differ in testosterone in white-faced marmosets (Birnie *et al*., 2011). Collectively, these findings suggest that testosterone does not reliably track breeder versus helper status in callitrichids, consistent with reduced male reproductive competition in cooperative breeders (Yamamoto *et al*., 2014).

Males exhibited higher testosterone concentrations than females across age classes and social roles. However, the magnitude of this sex difference was relatively small compared with patterns found in other species (Goymann & Wingfield, 2014; Wobber *et al*., 2013). For instance, mean testosterone values in chimpanzees are reported to be 14.6 times higher in males than females (Sonnweber *et al*., 2022), whereas in our marmoset sample male breeders averaged only 1.4-fold higher testosterone than female breeders (males: 46.3 pg/mg, standard deviation [SD] = 23.1; females: 33.4, SD = 14.8). Even bonobos (*Pan paniscus*), a species characterized by low intersexual aggression, show a 12.6-fold male–female testosterone ratio (Sonnweber *et al*., 2022)—a gap attributed largely to reduced male levels rather than elevated female levels (Sannen *et al*., 2003; Wobber *et al*., 2013). The comparatively modest sex difference in marmosets is consistent with evidence that female callitrichids can be highly competitive and assertive, particularly during intergroup encounters and in feeding contexts (Michels, 1998; Mustoe, 2023). Females also show significant testosterone increases in response to aggressive interactions, a pattern commonly observed in dominant males of other mammalian species (French *et al*., 2013; Ross & French, 2011). Together, these patterns raise the possibility that cooperative breeding and shared caregiving roles contribute to reduced sex differences in androgen profiles in common marmosets.

Consistent with developmental predictions, testosterone concentrations were low in pre-pubertal individuals and, in helpers of both sexes, increased with maturation and reached their peak in early adulthood. However, contrary to our prediction of a steady decline with age (Beehner *et al*., 2009; Ellison *et al*., 2002; Sorwell & Urbanski, 2013), testosterone levels in helpers remained broadly stable up to approximately eight years, the upper limit of the helper age range in our sample, and no clear age-related effects were detected in breeders or in the shared age-range dataset. This stability could reflect either preparedness for future breeding opportunities or maintenance of non-reproductive functions, but both interpretations are speculative and would require longitudinal data.

For androstenedione, the age pattern closely paralleled testosterone, consistent with androstenedione’s role as an adrenal- and gonadal-derived steroid and a biosynthetic precursor to testosterone (Mahesh & Greenblatt, 1962) and with comparable age-related patterns reported in humans (Bélanger *et al*., 1994; Davison *et al*., 2005). The apparent late-life deviation in older helpers should be interpreted cautiously given the small number of older helpers, but the strong covariation between androstenedione and testosterone (Supplementary Fig. 17) supports shared underlying endocrine regulation.

We detected a positive association between the presence of dependent offspring and testosterone concentrations in both the shared age-range and the breeders-only models. This contrasts with some reports of reduced testosterone around the immediate postpartum period in male marmosets based on serum or urinary measures (Nunes *et al*., 2000; Ziegler *et al*., 2009) and with broader evidence in humans linking fatherhood to lower testosterone (Gettler *et al*., 2011). However, endocrine responses to reproduction and caregiving are not uniform across callitrichids: in cotton-top tamarins (*Saguinus oedipus*), urinary testosterone has been reported to rise during a partner’s gestation and remain elevated postpartum (Ziegler & Snowdon, 2000). Similar variability has been reported in other biparental primates; for example, in red-bellied lemurs (*Eulemur rubriventer*), paternal infant-care investment was positively associated with fecal androgens during extended infant dependence (Tecot & Baden, 2018). One possibility is that testosterone decreases immediately postpartum but rebounds later during extended infant dependence, reflecting a shifting balance between caregiving demands and longer-term social or mating-related cues. Because our offspring predictor was defined at the group level and hair integrates endocrine output across months, our estimate may average across phase-specific postpartum dynamics that shorter-window callitrichid studies (typically focused on the first few weeks) cannot capture; thus, our approach cannot distinguish early postpartum changes from later rebounds within the integration window. Overall, testosterone was more strongly associated with sex and age than with breeding role, underscoring the need for longitudinal studies that link androgens to quantified caregiving and sociosexual behavior in cooperative breeders.

### Estradiol and Progesterone

Estradiol and progesterone showed partly dissociable patterns across breeding roles and sexes. Progesterone provided the clearest role-linked signal, expressed as a pronounced sex-by-role interaction: breeder females exhibited markedly elevated progesterone concentrations, whereas males remained consistently low across roles. This strong elevation in breeder females is consistent with progesterone tracking luteal activity and with the commonly reported reduction of ovulatory/luteal function in helper females in captive callitrichids, while acknowledging that the degree of reproductive suppression is context-dependent and can be incomplete in natal groups (Abbott & Hearn, 1978; Ziegler & Sousa, 2002). For estradiol, breeding role did not produce a consistent difference in the full dataset. Instead, a sex difference emerged within breeders, with female breeders higher than male breeders. Interpretation of ovarian steroids in breeder females should consider colony reproductive management. Breeder females received cloprostenol approximately every 6 weeks; given that hair integrates endocrine output over an average of about 8 months in our study, measured concentrations likely reflect multiple induced luteal regressions and subsequent follicular phases (Sotrel et al., 1981; Summers *et al*., 1985). Because luteolysis is expected to reduce luteal progesterone, elevated progesterone in breeder females is unlikely to be inflated by treatment and may instead be underestimated. Any influence on estradiol is indirect and timing-dependent and may primarily affect cycle timing and within-female variability rather than shifting long-term mean levels. However, even if cloprostenol has limited effects on long-term means, it imposed an artificial, persistently nonpregnant cycling state in female breeders that is unlikely to occur in this form in the wild.

We found age-related variation in both ovarian steroids, but the patterns were more pronounced for progesterone than for estradiol. Estradiol showed only modest age-related variation in helpers, suggesting that gonadal maturation and subsequent change can occur even in helper individuals and in the absence of breeding opportunities. This pattern is consistent with evidence that reproductive suppression is context-dependent in callitrichids (Ziegler & Sousa, 2002). Progesterone exhibited clearer age dependence. In helpers, progesterone varied with age without a statistically supported sex-by-age interaction, but the curvature appeared largely driven by females, with concentrations declining early and then increasing through adolescence, whereas males remained consistently low. The sex-specific pattern may have been obscured statistically by substantial within-group variation among females. In breeders, progesterone showed the expected female trajectory, increasing with age, peaking around eight years, and declining thereafter, while male breeder progesterone remained low and stable across adulthood, consistent with the relatively stable progesterone profiles reported for human males (Oettel & Mukhopadhyay, 2004).

The presence of dependent offspring in the group was not significantly associated with concentrations of estradiol or progesterone in our models. Reported links between estradiol and caregiving are variable in cooperative breeders. In females, maternal care has been associated with higher urinary estradiol in late-gestation/prepartum contexts in common marmosets and red-bellied tamarins (*Saguinus labiatus*) (Pryce, 1993; Pryce *et al*., 1988), whereas an inverse relationship was reported in black-tufted-ear marmosets (Fite & French, 2000). In callitrichids, postpartum ovulation and rapid resumption of cycling may attenuate lactation-associated suppression of ovarian steroids relative to many other primates (Dettmer *et al*., 2015; Kholkute, 1984; Shackleton, 1974; Ziegler *et al*., 1989), potentially limiting sustained infant-associated shifts in long-term estradiol and progesterone measures. In males, lower urinary estradiol has been linked to greater infant-care effort during late gestation and early postpartum in Wied’s marmosets (*Callithrix kuhlii*) (Nunes *et al*., 2000, 2001), whereas paternal-care variation was not associated with estradiol variation in white-faced marmosets (*Callithrix geoffroyi*) (Cavanaugh & French, 2013); elevated estradiol has also been reported during the expectant period and early fatherhood in cotton-top tamarins (Ziegler *et al*., 2004). Similar expectant-father increases occur in another biparental primate, red-bellied lemurs, where fecal estradiol metabolites rose during the mate’s pregnancy and then declined with infant age (Tecot *et al*., 2023). Together, these findings suggest stage-specific estradiol dynamics (expectant vs. early postpartum vs later infant dependence) that may not translate into sustained group-level differences across extended dependence. A group-level offspring predictor and a months-integrated hair measure may therefore average over peripartum shifts and individual variation in caregiving, obscuring detectable effects.

Overall, our findings provide a detailed account of long-term steroid hormone profiles in common marmosets, providing an integrated perspective on how endocrine patterns relate to cooperative breeding, social roles, and life-history traits. Our approach enabled us to detect stable, cumulative hormone patterns that are difficult to capture with more transient measures, while recognizing that some reproductive transitions may occur on shorter timescales and therefore require complementary sampling designs. Future work should assess whether similar patterns hold across other cooperatively breeding taxa. By integrating longitudinal designs and expanding cross-species comparisons, we can better understand how evolutionary pressures on caregiving, social cohesion, and reproductive coordination shape primate endocrinology.

## Supplementary Information

Below is the link to the electronic supplementary material.

Supplementary file1 (DOCX 4831 KB)
