# Supplementary material for: Hair Steroid Signatures of Cooperative Breeding in Male and Female Common Marmosets (Callithrix jacchus)
Source: Int J Primatol. 2026 May 23;47(4):988–1014. doi: 10.1007/s10764-026-00568-9 (PMC13661722; doi:10.1007/s10764-026-00568-9)
Supplement: Supplementary file 1 [file 10764_2026_568_MOESM1_ESM.docx]

**Supplementary materials**

**Table 1: Overview of analyzed hair samples.**
This table lists all samples included in the study, with columns indicating sample ID, date of collection, individual name, family group, sex, breeding role (breeder or helper), and the individual’s age in days at the time of sampling

| Sample_ID | Date | Name | Group | Sex | Breeding Role | Age |
| --- | --- | --- | --- | --- | --- | --- |
| 1 | 2021-11-19 | Nikita | Nikitas | f | breeder | 3414 |
| 2 | 2021-11-19 | Puk | Nikitas | m | breeder | 3383 |
| 3 | 2021-11-19 | Natsuki | Nikitas | f | helper | 1151 |
| 4 | 2021-11-19 | Nashi | Nikitas | f | helper | 1970 |
| 5 | 2021-11-19 | Nori | Nikitas | m | helper | 1970 |
| 6 | 2021-11-19 | Nyoko | Nikitas | f | helper | 1151 |
| 7 | 2021-11-19 | Nunchaku | Nikitas | m | helper | 2413 |
| 8 | 2021-12-01 | Grappa | Grappas | f | breeder | 2451 |
| 9 | 2021-12-01 | Craken | Grappas | m | breeder | 2974 |
| 10 | 2021-12-01 | Ginger | Grappas | f | helper | 1307 |
| 11 | 2021-12-01 | Giotto | Grappas | m | helper | 751 |
| 12 | 2021-12-01 | Gina | Grappas | f | helper | 751 |
| 13 | 2021-12-01 | Lima | Limas | f | breeder | 2481 |
| 14 | 2021-12-01 | Wolverine | Limas | m | breeder | 1301 |
| 15 | 2021-12-08 | Jaja | Jajas | f | breeder | 4484 |
| 16 | 2021-12-08 | Membo | Jajas | m | breeder | 4484 |
| 17 | 2021-12-08 | Jala | Jajas | f | helper | 2507 |
| 18 | 2021-12-08 | Jelly | Jajas | m | helper | 1986 |
| 19 | 2021-12-08 | Mibba | Mibbas | f | breeder | 4557 |
| 20 | 2021-12-08 | Conan | Mibbas | m | breeder | 2981 |
| 21 | 2021-12-08 | Madame | Mibbas | f | helper | 1340 |
| 22 | 2021-12-08 | Madita | Mibbas | f | helper | 1340 |
| 23 | 2021-12-09 | Jumbo | Jandiras | m | helper | 438 |
| 24 | 2021-12-09 | Guarana | Guapas | f | helper | 279 |
| 25 | 2021-12-09 | Gimli | Guapas | m | helper | 279 |
| 28 | 2021-12-15 | Washington | Washingtons | f | breeder | 3029 |
| 29 | 2021-12-15 | Lotus | Washingtons | m | breeder | 3452 |
| 31 | 2021-12-15 | Lancia | Lancias | f | breeder | 7051 |
| 32 | 2021-12-15 | Lexus | Lancias | m | breeder | 6724 |
| 33 | 2021-12-15 | Lola | Lancias | f | helper | 3183 |
| 34 | 2021-12-22 | Jandira | Jandiras | f | breeder | 2521 |
| 35 | 2021-12-22 | Wombat | Jandiras | m | breeder | 1803 |
| 36 | 2021-12-22 | Guapa | Guapas | f | breeder | 1328 |
| 37 | 2021-12-22 | Ninja | Guapas | m | breeder | 2446 |
| 38 | 2021-12-22 | Wasabi | Wasabis | f | breeder | 1803 |
| 39 | 2021-12-22 | Lynx | Wasabis | m | breeder | 1125 |
| 40 | 2021-12-29 | Jambi | Jambis | f | breeder | 2241 |
| 41 | 2021-12-29 | Werewolf | Jambis | m | breeder | 1329 |
| 42 | 2022-03-02 | Wuschel | Ex Wisconsin | m | helper | 861 |
| 43 | 2022-03-02 | Lex | Ex Ninas | m | breeder | 5712 |
| 44 | 2022-03-02 | Nougat | Ex Ninas | f | helper | 2513 |
| 45 | 2022-03-02 | Nox | Ex Ninas | m | helper | 2068 |
| 46 | 2022-03-02 | Nux | Ex Ninas | f | helper | 2067 |
| 47 | 2022-03-09 | Nala | Nalas | f | breeder | 4636 |
| 48 | 2022-03-09 | Nasa | Nalas | f | helper | 842 |
| 49 | 2022-03-09 | Nemo | Nalas | m | helper | 842 |
| 50 | 2022-03-09 | Nirvana | Nalas | f | helper | 2664 |
| 51 | 2022-04-06 | Nugatti | Nugattis | f | breeder | 870 |
| 52 | 2022-04-06 | Odin | Nugattis | m | breeder | 2232 |
| 53 | 2022-04-06 | Lima | Limas | f | breeder | 2607 |
| 54 | 2022-04-06 | Jam | Ex Nutellas | m | breeder | 2105 |
| 55 | 2022-04-06 | Nocioletta | Ex Nutellas | f | helper | 870 |
| 59 | 2022-04-13 | Merkur | Jupies | m | breeder | 3563 |
| 60 | 2022-04-13 | Jupie | Jupies | f | breeder | 4610 |
| 61 | 2022-04-20 | Lea | Leas | f | breeder | 5359 |
| 62 | 2022-04-20 | Kapi | Leas | m | breeder | 7171 |
| 67 | 2022-05-11 | Mulan | Mulans | f | breeder | 1811 |
| 68 | 2022-05-11 | Nautilus | Mulans | m | breeder | 3430 |
| 72 | 2022-06-29 | Wall-e | Washingtons | f | helper | 321 |
| 73 | 2022-06-29 | Woody | Washingtons | m | helper | 321 |
| 74 | 2022-06-29 | Lima | Limas | f | breeder | 2691 |
| 75 | 2022-06-29 | Lychee | Limas | f | helper | 252 |
| 76 | 2022-06-29 | Lemon | Limas | m | helper | 252 |
| 78 | 2022-06-29 | Wombat | Jandiras | m | breeder | 1992 |
| 79 | 2022-06-29 | Jumbo | Jandiras | m | helper | 640 |
| 81 | 2022-07-13 | Washington | Washingtons | f | breeder | 3239 |
| 82 | 2022-07-13 | Lotus | Washingtons | m | breeder | 3662 |
| 83 | 2022-07-13 | Grappa | Grappas | f | breeder | 2675 |
| 84 | 2022-07-13 | Craken | Grappas | m | breeder | 3198 |
| 85 | 2022-07-13 | Giotto | Grappas | m | helper | 975 |
| 86 | 2022-07-13 | Gina | Grappas | f | helper | 975 |
| 87 | 2022-07-20 | Wasabi | Wasabis | f | breeder | 2013 |
| 88 | 2022-07-20 | Lynx | Wasabis | m | breeder | 1335 |
| 89 | 2022-07-20 | Wonton | Wasabis | f | helper | 497 |
| 90 | 2022-07-20 | Wakame | Wasabis | f | helper | 497 |
| 91 | 2022-07-20 | Guapa | Guapas | f | breeder | 1538 |
| 92 | 2022-07-20 | Mibba | Mibbas | f | breeder | 4781 |
| 93 | 2022-07-20 | Conan | Mibbas | m | breeder | 3205 |
| 94 | 2022-07-20 | Madame | Mibbas | f | helper | 1564 |
| 95 | 2022-07-20 | Madita | Mibbas | f | helper | 1564 |
| 96 | 2022-07-27 | Nikita | Nikitas | f | breeder | 3664 |
| 97 | 2022-07-27 | Nyoko | Nikitas | f | helper | 1401 |
| 98 | 2022-07-27 | Natsuki | Nikitas | f | helper | 1401 |
| 99 | 2022-07-27 | Puk | Nikitas | m | breeder | 3633 |
| 100 | 2022-07-27 | Nunchaku | Nikitas | m | helper | 2663 |
| 101 | 2022-07-27 | Nori | Nikitas | m | helper | 2220 |
| 102 | 2022-07-27 | Nashi | Nikitas | f | helper | 2220 |
| 103 | 2022-07-27 | Jambi | Jambis | f | breeder | 2451 |
| 104 | 2022-07-27 | Werewolf | Jambis | m | breeder | 1539 |
| 105 | 2022-07-27 | Jafar | Jambis | m | helper | 385 |
| 106 | 2022-07-27 | Jaggar | Jambis | m | helper | 385 |
| 107 | 2022-08-03 | Jaja | Jajas | f | breeder | 4722 |
| 108 | 2022-08-03 | Membo | Jajas | m | breeder | 4722 |
| 109 | 2022-08-03 | Jala | Jajas | f | helper | 2745 |
| 110 | 2022-08-03 | Jelly | Jajas | m | helper | 2224 |
| 111 | 2022-08-10 | Wolverine | Limas | m | breeder | 1553 |
| 114 | 2023-01-04 | Guapa | Guapas | f | breeder | 1706 |
| 115 | 2023-02-01 | Lola | Lolas | f | breeder | 3596 |
| 116 | 2023-02-08 | Washington | Washingtons | f | breeder | 3449 |
| 117 | 2023-02-08 | Lotus | Washingtons | m | breeder | 3872 |
| 118 | 2023-02-08 | Woody | Washingtons | m | helper | 545 |
| 119 | 2023-02-08 | Wall-e | Washingtons | f | helper | 545 |
| 120 | 2023-02-16 | Lynx | Wasabis | m | breeder | 1546 |
| 121 | 2023-02-16 | Wakame | Wasabis | f | helper | 708 |
| 122 | 2023-02-16 | Wonton | Wasabis | f | helper | 708 |
| 123 | 2023-02-16 | Jaja | Jajas | f | breeder | 4919 |
| 124 | 2023-02-23 | Membo | Jajas | m | breeder | 4926 |
| 125 | 2023-02-23 | Jala | Jajas | f | helper | 2949 |
| 126 | 2023-02-22 | Grappa | Grappas | f | breeder | 2899 |
| 127 | 2023-02-22 | Gina | Grappas | f | helper | 1199 |
| 128 | 2023-02-22 | Giotto | Grappas | m | helper | 1199 |
| 131 | 2023-03-08 | Lex | Ex Ninas | m | breeder | 6083 |
| 132 | 2023-03-08 | Nougat | Ex Ninas | f | helper | 2884 |
| 133 | 2023-03-08 | Nox | Ex Ninas | m | helper | 2439 |
| 134 | 2023-03-08 | Lima | Limas | f | breeder | 2943 |
| 135 | 2023-03-08 | Wolverine | Limas | m | breeder | 1763 |
| 136 | 2023-03-08 | Lemon | Limas | m | helper | 504 |
| 137 | 2023-03-08 | Lychee | Limas | f | helper | 504 |
| 139 | 2023-03-15 | Jumbo | Jandiras | m | helper | 899 |
| 140 | 2023-03-15 | Wombat | Jandiras | m | breeder | 2251 |
| 141 | 2023-03-15 | Tabor | Lolas | m | breeder | 5249 |
| 142 | 2023-04-05 | Jambi | Jambis | f | breeder | 2703 |
| 143 | 2023-04-05 | Jaggar | Jambis | m | helper | 637 |
| 144 | 2023-04-05 | Jafar | Jambis | m | helper | 637 |
| 145 | 2023-04-05 | Werewolf | Jambis | m | breeder | 1791 |
| 146 | 2023-04-12 | Jam | Gingers | m | breeder | 2476 |
| 152 | 2023-05-10 | Gimli | Guapas | m | helper | 796 |
| 153 | 2023-05-10 | Ninja | Guapas | m | breeder | 2950 |
| 154 | 2023-05-10 | Guarana | Guapas | f | helper | 796 |
| 157 | 2023-06-07 | Lancia | Lancias | f | breeder | 7590 |
| 158 | 2023-06-07 | Lexus | Lancias | m | breeder | 7263 |
| 165 | 2023-07-05 | Jupie | Jupies | f | breeder | 5058 |
| 166 | 2023-07-05 | Merkur | Jupies | m | breeder | 4011 |
| 167 | 2023-07-10 | Jambi | Jambis | f | breeder | 2799 |
| 168 | 2023-07-10 | Jaggar | Jambis | m | helper | 733 |
| 169 | 2023-07-10 | Jafar | Jambis | m | helper | 733 |
| 170 | 2023-07-10 | Werewolf | Jambis | m | breeder | 1887 |
| 171 | 2023-07-11 | Guapa | Guapas | f | breeder | 1894 |
| 172 | 2023-07-11 | Ninja | Guapas | m | breeder | 3012 |
| 173 | 2023-07-11 | Guarana | Guapas | f | helper | 858 |
| 174 | 2023-07-11 | Gimli | Guapas | m | helper | 858 |
| 175 | 2023-07-12 | Wuschel | Olympias | m | breeder | 1358 |
| 176 | 2023-07-12 | Odessa | Olympias | f | helper | 215 |
| 177 | 2023-07-12 | Ocarina | Olympias | f | helper | 215 |
| 178 | 2023-07-12 | Lima | Limas | f | breeder | 3069 |
| 179 | 2023-07-12 | Lemon | Limas | m | helper | 630 |
| 180 | 2023-07-12 | Lychee | Limas | f | helper | 630 |
| 184 | 2023-07-19 | Jandira | Jandiras | f | breeder | 3095 |
| 185 | 2023-07-19 | Wombat | Jandiras | m | breeder | 2377 |
| 186 | 2023-07-19 | Juba | Jandiras | f | helper | 253 |
| 187 | 2023-07-19 | Jumbo | Jandiras | m | helper | 1025 |
| 188 | 2023-07-19 | Jamba | Jandiras | f | helper | 253 |
| 189 | 2023-07-26 | Washington | Washingtons | f | breeder | 3617 |
| 190 | 2023-07-23 | Lotus | Washingtons | m | breeder | 4037 |
| 191 | 2023-07-23 | Wall-e | Washingtons | f | helper | 710 |
| 192 | 2023-07-23 | Woody | Washingtons | m | helper | 710 |
| 193 | 2023-08-02 | Wasabi | Wasabis | f | breeder | 2391 |
| 194 | 2023-08-09 | Grappa | Grappas | f | breeder | 3067 |
| 195 | 2023-08-09 | Craken | Grappas | m | breeder | 3590 |
| 196 | 2023-08-09 | Giotto | Grappas | m | helper | 1367 |
| 197 | 2023-08-09 | Gina | Grappas | f | helper | 1367 |
| 198 | 2023-08-09 | Mibba | Mibbas | f | breeder | 5166 |
| 199 | 2023-08-09 | Conan | Mibbas | m | breeder | 3590 |
| 200 | 2023-08-09 | Madita | Mibbas | f | helper | 1949 |
| 201 | 2023-08-09 | Madame | Mibbas | f | helper | 1949 |
| 204 | 2023-11-22 | Lola | Lolas | f | breeder | 3890 |
| 205 | 2023-11-22 | Tabor | Lolas | m | breeder | 5501 |
| 206 | 2023-11-22 | Jandira | Jandiras | f | breeder | 3221 |
| 207 | 2023-11-22 | Wombat | Jandiras | m | breeder | 2503 |
| 208 | 2023-11-22 | Juba | Jandiras | f | helper | 379 |
| 209 | 2023-11-22 | Jamba | Jandiras | f | helper | 379 |
| 210 | 2023-11-22 | Olympia | Olympias | f | breeder | 1878 |
| 211 | 2023-11-22 | Wuschel | Olympias | m | breeder | 1491 |
| 212 | 2023-11-22 | Ocarina | Olympias | f | helper | 348 |
| 215 | 2023-11-29 | Washington | Washingtons | f | breeder | 3743 |
| 216 | 2023-11-29 | Lotus | Washingtons | m | breeder | 4166 |
| 217 | 2023-11-29 | Wall-e | Washingtons | f | helper | 839 |
| 218 | 2023-11-29 | Woody | Washingtons | m | helper | 839 |
| 220 | 2023-12-06 | Lynx | Wasabis | m | breeder | 1839 |
| 221 | 2023-12-06 | Wonton | Wasabis | f | helper | 1001 |
| 222 | 2023-12-06 | Wakame | Wasabis | f | helper | 1001 |
| 224 | 2023-12-06 | Guapa | Guapas | f | breeder | 2042 |
| 225 | 2023-12-06 | Ninja | Guapas | m | breeder | 3160 |
| 226 | 2023-12-06 | Guarana | Guapas | f | helper | 1006 |
| 228 | 2023-12-13 | Gina | Grappas | f | helper | 1493 |
| 229 | 2023-12-13 | Craken | Grappas | m | breeder | 3716 |
| 230 | 2023-12-13 | Jambi | Jambis | f | breeder | 2955 |
| 231 | 2023-12-13 | Werewolf | Jambis | m | breeder | 2043 |
| 232 | 2023-12-13 | Jafar | Jambis | m | helper | 889 |
| 233 | 2023-12-13 | Jaggar | Jambis | m | helper | 889 |
| 234 | 2023-12-27 | Lima | Limas | f | breeder | 3237 |
| 235 | 2023-12-27 | Wolverine | Limas | m | breeder | 2057 |
| 236 | 2023-12-27 | Lychee | Limas | f | helper | 798 |

**Washing test**

These tables show hormone concentrations (ng/ml) measured in the isopropanol solutions collected during a washing test conducted on 10 hair samples (5 males, 5 females). For each sample, four consecutive 20-second washes were performed with 2 ml of isopropanol. After each wash, the isopropanol was recovered and analyzed separately to assess potential hormone loss from the hair. The table includes the hormone name, sample ID, wash number (1–4), and the concentration measured in each washing solution.

The results show that most detectable hormone residues were present in the first washing solution, while hormone concentrations in subsequent washes were minimal or below detection limits for most hormones. This indicates that a single, brief isopropanol wash is sufficient to remove surface contaminants with minimal risk of extracting hormones from within the hair shaft.

*Table 2: Glucocorticoids*

| Hormone | Sample | Wash Number | | ng/ml |  |
| --- | --- | --- | --- | --- | --- |
| Cortisol | 20 | Washing solution #1 | | 3.339467 |  |
| Cortisol | 20 | Washing solution #2 | | 0.917422 |  |
| Cortisol | 20 | Washing solution #3 | | 0 |  |
| Cortisol | 20 | Washing solution #4 | | 0 |  |
| Cortisol | 22 | Washing solution #1 | | 5.6256 |  |
| Cortisol | 22 | Washing solution #2 | | 1.4784 |  |
| Cortisol | 22 | Washing solution #3 | | 0.652978 |  |
| Cortisol | 22 | Washing solution #4 | | 0.451822 |  |
| Cortisol | 35 | Washing solution #1 | | 7.955911 |  |
| Cortisol | 35 | Washing solution #2 | | 3.321956 |  |
| Cortisol | 35 | Washing solution #3 | | 0.813778 |  |
| Cortisol | 35 | Washing solution #4 | | 0 |  |
| Cortisol | 40 | Washing solution #1 | | 9.207911 |  |
| Cortisol | 40 | Washing solution #2 | | 2.02 |  |
| Cortisol | 40 | Washing solution #3 | | 0.850044 |  |
| Cortisol | 40 | Washing solution #4 | | 0 |  |
| Cortisol | 47 | Washing solution #1 | | 5.697067 |  |
| Cortisol | 47 | Washing solution #2 | | 0.878489 |  |
| Cortisol | 47 | Washing solution #3 | | 0 |  |
| Cortisol | 47 | Washing solution #4 | | 0 |  |
| Cortisol | 68 | Washing solution #1 | | 3.080356 |  |
| Cortisol | 68 | Washing solution #2 | | 0 |  |
| Cortisol | 68 | Washing solution #3 | | 0 |  |
| Cortisol | 68 | Washing solution #4 | | 0 |  |
| Cortisol | 83 | Washing solution #1 | | 15.32293 |  |
| Cortisol | 83 | Washing solution #2 | | 0 |  |
| Cortisol | 83 | Washing solution #3 | | 0 |  |
| Cortisol | 83 | Washing solution #4 | | 0 |  |
| Cortisol | 88 | Washing solution #1 | | 9.162222 |  |
| Cortisol | 88 | Washing solution #2 | | 0.764178 |  |
| Cortisol | 88 | Washing solution #3 | | 0 |  |
| Cortisol | 88 | Washing solution #4 | | 0 |  |
| Cortisol | 80 | Washing solution #1 | | 2.1841 |  |
| Cortisol | 80 | Washing solution #2 | | 1.112089 |  |
| Cortisol | 80 | Washing solution #3 | | 0 |  |
| Cortisol | 80 | Washing solution #4 | | 0 |  |
| Cortisol | 86 | Washing solution #1 | | 5.767022 |  |
| Cortisol | 86 | Washing solution #2 | | 0 |  |
| Cortisol | 86 | Washing solution #3 | | 0 |  |
| Cortisol | 86 | Washing solution #4 | | 0 |  |
| Corticosterone | 20 | Washing solution #1 | 0 | | |
| Corticosterone | 20 | Washing solution #2 | 0 | | |
| Corticosterone | 20 | Washing solution #3 | 0 | | |
| Corticosterone | 20 | Washing solution #4 | 0 | | |
| Corticosterone | 22 | Washing solution #1 | 0 | | |
| Corticosterone | 22 | Washing solution #2 | 0 | | |
| Corticosterone | 22 | Washing solution #3 | 0 | | |
| Corticosterone | 22 | Washing solution #4 | 0 | | |
| Corticosterone | 35 | Washing solution #1 | 0 | | |
| Corticosterone | 35 | Washing solution #2 | 0 | | |
| Corticosterone | 35 | Washing solution #3 | 0 | | |
| Corticosterone | 35 | Washing solution #4 | 0 | | |
| Corticosterone | 40 | Washing solution #1 | 0.167644 | | |
| Corticosterone | 40 | Washing solution #2 | 0 | | |
| Corticosterone | 40 | Washing solution #3 | 0 | | |
| Corticosterone | 40 | Washing solution #4 | 0 | | |
| Corticosterone | 47 | Washing solution #1 | 0.224 | | |
| Corticosterone | 47 | Washing solution #2 | 0 | | |
| Corticosterone | 47 | Washing solution #3 | 0 | | |
| Corticosterone | 47 | Washing solution #4 | 0 | | |
| Corticosterone | 68 | Washing solution #1 | 0 | | |
| Corticosterone | 68 | Washing solution #2 | 0 | | |
| Corticosterone | 68 | Washing solution #3 | 0 | | |
| Corticosterone | 68 | Washing solution #4 | 0 | | |
| Corticosterone | 83 | Washing solution #1 | 0 | | |
| Corticosterone | 83 | Washing solution #2 | 0 | | |
| Corticosterone | 83 | Washing solution #3 | 0 | | |
| Corticosterone | 83 | Washing solution #4 | 0 | | |
| Corticosterone | 88 | Washing solution #1 | 0 | | |
| Corticosterone | 88 | Washing solution #2 | 0 | | |
| Corticosterone | 88 | Washing solution #3 | 0 | | |
| Corticosterone | 88 | Washing solution #4 | 0 | | |
| Corticosterone | 80 | Washing solution #1 | 0 | | |
| Corticosterone | 80 | Washing solution #2 | 0 | | |
| Corticosterone | 80 | Washing solution #3 | 0 | | |
| Corticosterone | 80 | Washing solution #4 | 0 | | |
| Corticosterone | 86 | Washing solution #1 | 0 | | |
| Corticosterone | 86 | Washing solution #2 | 0 | | |
| Corticosterone | 86 | Washing solution #3 | 0 | | |
| Corticosterone | 86 | Washing solution #4 | 0 | | |
| Cortisone | 20 | Washing solution #1 | 0.610133 | | |
| Cortisone | 20 | Washing solution #2 | 0 | | |
| Cortisone | 20 | Washing solution #3 | 0 | | |
| Cortisone | 20 | Washing solution #4 | 0 | | |
| Cortisone | 22 | Washing solution #1 | 1.722578 | | |
| Cortisone | 22 | Washing solution #2 | 0 | | |
| Cortisone | 22 | Washing solution #3 | 0 | | |
| Cortisone | 22 | Washing solution #4 | 0 | | |
| Cortisone | 35 | Washing solution #1 | 0.898756 | | |
| Cortisone | 35 | Washing solution #2 | 0 | | |
| Cortisone | 35 | Washing solution #3 | 0 | | |
| Cortisone | 35 | Washing solution #4 | 0 | | |
| Cortisone | 40 | Washing solution #1 | 2.170222 | | |
| Cortisone | 40 | Washing solution #2 | 0 | | |
| Cortisone | 40 | Washing solution #3 | 0 | | |
| Cortisone | 40 | Washing solution #4 | 0 | | |
| Cortisone | 47 | Washing solution #1 | 1.065422 | | |
| Cortisone | 47 | Washing solution #2 | 0 | | |
| Cortisone | 47 | Washing solution #3 | 0 | | |
| Cortisone | 47 | Washing solution #4 | 0 | | |
| Cortisone | 68 | Washing solution #1 | 0.711378 | | |
| Cortisone | 68 | Washing solution #2 | 0 | | |
| Cortisone | 68 | Washing solution #3 | 0 | | |
| Cortisone | 68 | Washing solution #4 | 0 | | |
| Cortisone | 83 | Washing solution #1 | 9.044089 | | |
| Cortisone | 83 | Washing solution #2 | 0 | | |
| Cortisone | 83 | Washing solution #3 | 0 | | |
| Cortisone | 83 | Washing solution #4 | 0 | | |
| Cortisone | 88 | Washing solution #1 | 2.9088 | | |
| Cortisone | 88 | Washing solution #2 | 0 | | |
| Cortisone | 88 | Washing solution #3 | 0 | | |
| Cortisone | 88 | Washing solution #4 | 0 | | |
| Cortisone | 80 | Washing solution #1 | 2.1841 | | |
| Cortisone | 80 | Washing solution #2 | 0 | | |
| Cortisone | 80 | Washing solution #3 | 0 | | |
| Cortisone | 80 | Washing solution #4 | 0 | | |
| Cortisone | 86 | Washing solution #1 | 2.074933 | | |
| Cortisone | 86 | Washing solution #2 | 0 | | |
| Cortisone | 86 | Washing solution #3 | 0 | | |
| Cortisone | 86 | Washing solution #4 | 0 | | |

*Table 3: Androgens*

| Hormone | Sample | Wash Number | ng/ml |
| --- | --- | --- | --- |
| Testosterone | 20 | Washing solution #1 | 0 |
| Testosterone | 20 | Washing solution #2 | 0 |
| Testosterone | 20 | Washing solution #3 | 0 |
| Testosterone | 20 | Washing solution #4 | 0 |
| Testosterone | 22 | Washing solution #1 | 0 |
| Testosterone | 22 | Washing solution #2 | 0 |
| Testosterone | 22 | Washing solution #3 | 0 |
| Testosterone | 22 | Washing solution #4 | 0 |
| Testosterone | 35 | Washing solution #1 | 0 |
| Testosterone | 35 | Washing solution #2 | 0 |
| Testosterone | 35 | Washing solution #3 | 0 |
| Testosterone | 35 | Washing solution #4 | 0 |
| Testosterone | 40 | Washing solution #1 | 0 |
| Testosterone | 40 | Washing solution #2 | 0 |
| Testosterone | 40 | Washing solution #3 | 0 |
| Testosterone | 40 | Washing solution #4 | 0 |
| Testosterone | 47 | Washing solution #1 | 0 |
| Testosterone | 47 | Washing solution #2 | 0 |
| Testosterone | 47 | Washing solution #3 | 0 |
| Testosterone | 47 | Washing solution #4 | 0 |
| Testosterone | 68 | Washing solution #1 | 0 |
| Testosterone | 68 | Washing solution #2 | 0 |
| Testosterone | 68 | Washing solution #3 | 0 |
| Testosterone | 68 | Washing solution #4 | 0 |
| Testosterone | 83 | Washing solution #1 | 0 |
| Testosterone | 83 | Washing solution #2 | 0 |
| Testosterone | 83 | Washing solution #3 | 0 |
| Testosterone | 83 | Washing solution #4 | 0 |
| Testosterone | 88 | Washing solution #1 | 0 |
| Testosterone | 88 | Washing solution #2 | 0 |
| Testosterone | 88 | Washing solution #3 | 0 |
| Testosterone | 88 | Washing solution #4 | 0 |
| Testosterone | 80 | Washing solution #1 | 0 |
| Testosterone | 80 | Washing solution #2 | 0 |
| Testosterone | 80 | Washing solution #3 | 0 |
| Testosterone | 80 | Washing solution #4 | 0 |
| Testosterone | 86 | Washing solution #1 | 0 |
| Testosterone | 86 | Washing solution #2 | 0 |
| Testosterone | 86 | Washing solution #3 | 0 |
| Testosterone | 86 | Washing solution #4 | 0 |
| Androstenedione | 20 | Washing solution #1 | 0 |
| Androstenedione | 20 | Washing solution #2 | 0 |
| Androstenedione | 20 | Washing solution #3 | 0 |
| Androstenedione | 20 | Washing solution #4 | 0 |
| Androstenedione | 22 | Washing solution #1 | 0 |
| Androstenedione | 22 | Washing solution #2 | 0 |
| Androstenedione | 22 | Washing solution #3 | 0 |
| Androstenedione | 22 | Washing solution #4 | 0 |
| Androstenedione | 35 | Washing solution #1 | 0 |
| Androstenedione | 35 | Washing solution #2 | 0 |
| Androstenedione | 35 | Washing solution #3 | 0 |
| Androstenedione | 35 | Washing solution #4 | 0 |
| Androstenedione | 40 | Washing solution #1 | 0 |
| Androstenedione | 40 | Washing solution #2 | 0 |
| Androstenedione | 40 | Washing solution #3 | 0 |
| Androstenedione | 40 | Washing solution #4 | 0 |
| Androstenedione | 47 | Washing solution #1 | 0.110489 |
| Androstenedione | 47 | Washing solution #2 | 0 |
| Androstenedione | 47 | Washing solution #3 | 0 |
| Androstenedione | 47 | Washing solution #4 | 0 |
| Androstenedione | 68 | Washing solution #1 | 0 |
| Androstenedione | 68 | Washing solution #2 | 0 |
| Androstenedione | 68 | Washing solution #3 | 0 |
| Androstenedione | 68 | Washing solution #4 | 0 |
| Androstenedione | 83 | Washing solution #1 | 0.242489 |
| Androstenedione | 83 | Washing solution #2 | 0 |
| Androstenedione | 83 | Washing solution #3 | 0 |
| Androstenedione | 83 | Washing solution #4 | 0 |
| Androstenedione | 88 | Washing solution #1 | 0.116267 |
| Androstenedione | 88 | Washing solution #2 | 0 |
| Androstenedione | 88 | Washing solution #3 | 0 |
| Androstenedione | 88 | Washing solution #4 | 0 |
| Androstenedione | 80 | Washing solution #1 | 0 |
| Androstenedione | 80 | Washing solution #2 | 0 |
| Androstenedione | 80 | Washing solution #3 | 0 |
| Androstenedione | 80 | Washing solution #4 | 0 |
| Androstenedione | 86 | Washing solution #1 | 0.255378 |
| Androstenedione | 86 | Washing solution #2 | 0 |
| Androstenedione | 86 | Washing solution #3 | 0 |
| Androstenedione | 86 | Washing solution #4 | 0 |
| 11-ketotestosterone | 20 | Washing solution #1 | 0 |
| 11-ketotestosterone | 20 | Washing solution #2 | 0 |
| 11-ketotestosterone | 20 | Washing solution #3 | 0 |
| 11-ketotestosterone | 20 | Washing solution #4 | 0 |
| 11-ketotestosterone | 22 | Washing solution #1 | 0 |
| 11-ketotestosterone | 22 | Washing solution #2 | 0 |
| 11-ketotestosterone | 22 | Washing solution #3 | 0 |
| 11-ketotestosterone | 22 | Washing solution #4 | 0 |
| 11-ketotestosterone | 35 | Washing solution #1 | 0 |
| 11-ketotestosterone | 35 | Washing solution #2 | 0 |
| 11-ketotestosterone | 35 | Washing solution #3 | 0 |
| 11-ketotestosterone | 35 | Washing solution #4 | 0 |
| 11-ketotestosterone | 40 | Washing solution #1 | 0 |
| 11-ketotestosterone | 40 | Washing solution #2 | 0 |
| 11-ketotestosterone | 40 | Washing solution #3 | 0 |
| 11-ketotestosterone | 40 | Washing solution #4 | 0 |
| 11-ketotestosterone | 47 | Washing solution #1 | 0 |
| 11-ketotestosterone | 47 | Washing solution #2 | 0 |
| 11-ketotestosterone | 47 | Washing solution #3 | 0 |
| 11-ketotestosterone | 47 | Washing solution #4 | 0 |
| 11-ketotestosterone | 68 | Washing solution #1 | 0 |
| 11-ketotestosterone | 68 | Washing solution #2 | 0 |
| 11-ketotestosterone | 68 | Washing solution #3 | 0 |
| 11-ketotestosterone | 68 | Washing solution #4 | 0 |
| 11-ketotestosterone | 83 | Washing solution #1 | 0 |
| 11-ketotestosterone | 83 | Washing solution #2 | 0 |
| 11-ketotestosterone | 83 | Washing solution #3 | 0 |
| 11-ketotestosterone | 83 | Washing solution #4 | 0 |
| 11-ketotestosterone | 88 | Washing solution #1 | 0 |
| 11-ketotestosterone | 88 | Washing solution #2 | 0 |
| 11-ketotestosterone | 88 | Washing solution #3 | 0 |
| 11-ketotestosterone | 88 | Washing solution #4 | 0 |
| 11-ketotestosterone | 80 | Washing solution #1 | 0 |
| 11-ketotestosterone | 80 | Washing solution #2 | 0 |
| 11-ketotestosterone | 80 | Washing solution #3 | 0 |
| 11-ketotestosterone | 80 | Washing solution #4 | 0 |
| 11-ketotestosterone | 86 | Washing solution #1 | 0 |
| 11-ketotestosterone | 86 | Washing solution #2 | 0 |
| 11-ketotestosterone | 86 | Washing solution #3 | 0 |
| 11-ketotestosterone | 86 | Washing solution #4 | 0 |

*Table 4: Estradiol & Progesterone*

| Hormone | Sample | Wash Number | ng/ml |
| --- | --- | --- | --- |
| Estradiol | 20 | Washing solution #1 | 17.2848 |
| Estradiol | 20 | Washing solution #2 | 1.48648889 |
| Estradiol | 20 | Washing solution #3 | 0.35386667 |
| Estradiol | 20 | Washing solution #4 | 0.11075556 |
| Estradiol | 22 | Washing solution #1 | 31.0443556 |
| Estradiol | 22 | Washing solution #2 | 2.81511111 |
| Estradiol | 22 | Washing solution #3 | 1.29608889 |
| Estradiol | 22 | Washing solution #4 | 0.49502222 |
| Estradiol | 35 | Washing solution #1 | 8.16915556 |
| Estradiol | 35 | Washing solution #2 | 1.3768 |
| Estradiol | 35 | Washing solution #3 | 0.18764444 |
| Estradiol | 35 | Washing solution #4 | 0.14186667 |
| Estradiol | 40 | Washing solution #1 | 17.5062222 |
| Estradiol | 40 | Washing solution #2 | 1.82995556 |
| Estradiol | 40 | Washing solution #3 | 0.4408 |
| Estradiol | 40 | Washing solution #4 | 0.18062222 |
| Estradiol | 47 | Washing solution #1 | 13.9813333 |
| Estradiol | 47 | Washing solution #2 | 0.65084444 |
| Estradiol | 47 | Washing solution #3 | 0.07484444 |
| Estradiol | 47 | Washing solution #4 | 0.08417778 |
| Estradiol | 68 | Washing solution #1 | 7.88426667 |
| Estradiol | 68 | Washing solution #2 | 0.49751111 |
| Estradiol | 68 | Washing solution #3 | 0.13688889 |
| Estradiol | 68 | Washing solution #4 | 0 |
| Estradiol | 83 | Washing solution #1 | 41.3352889 |
| Estradiol | 83 | Washing solution #2 | 0.54977778 |
| Estradiol | 83 | Washing solution #3 | 0.33208889 |
| Estradiol | 83 | Washing solution #4 | 0 |
| Estradiol | 88 | Washing solution #1 | 9.39253333 |
| Estradiol | 88 | Washing solution #2 | 0.35475556 |
| Estradiol | 88 | Washing solution #3 | 0.1144 |
| Estradiol | 88 | Washing solution #4 | 0 |
| Estradiol | 80 | Washing solution #1 | 12.6025778 |
| Estradiol | 80 | Washing solution #2 | 0.61475556 |
| Estradiol | 80 | Washing solution #3 | 0.18613333 |
| Estradiol | 80 | Washing solution #4 | 0.08515556 |
| Estradiol | 86 | Washing solution #1 | 25.0026667 |
| Estradiol | 86 | Washing solution #2 | 0.55582222 |
| Estradiol | 86 | Washing solution #3 | 0.41093333 |
| Estradiol | 86 | Washing solution #4 | 0.12328889 |
| Progesterone | 20 | Washing solution #1 | 0.419378 |
| Progesterone | 20 | Washing solution #2 | 0 |
| Progesterone | 20 | Washing solution #3 | 0 |
| Progesterone | 20 | Washing solution #4 | 0 |
| Progesterone | 22 | Washing solution #1 | 0.368622 |
| Progesterone | 22 | Washing solution #2 | 0 |
| Progesterone | 22 | Washing solution #3 | 0 |
| Progesterone | 22 | Washing solution #4 | 0 |
| Progesterone | 35 | Washing solution #1 | 0.214044 |
| Progesterone | 35 | Washing solution #2 | 0 |
| Progesterone | 35 | Washing solution #3 | 0 |
| Progesterone | 35 | Washing solution #4 | 0 |
| Progesterone | 40 | Washing solution #1 | 0.635022 |
| Progesterone | 40 | Washing solution #2 | 0 |
| Progesterone | 40 | Washing solution #3 | 0 |
| Progesterone | 40 | Washing solution #4 | 0 |
| Progesterone | 47 | Washing solution #1 | 0.731378 |
| Progesterone | 47 | Washing solution #2 | 0 |
| Progesterone | 47 | Washing solution #3 | 0 |
| Progesterone | 47 | Washing solution #4 | 0 |
| Progesterone | 68 | Washing solution #1 | 0.206489 |
| Progesterone | 68 | Washing solution #2 | 0 |
| Progesterone | 68 | Washing solution #3 | 0 |
| Progesterone | 68 | Washing solution #4 | 0 |
| Progesterone | 83 | Washing solution #1 | 4.995467 |
| Progesterone | 83 | Washing solution #2 | 0.032444 |
| Progesterone | 83 | Washing solution #3 | 0 |
| Progesterone | 83 | Washing solution #4 | 0 |
| Progesterone | 88 | Washing solution #1 | 0.5136 |
| Progesterone | 88 | Washing solution #2 | 0 |
| Progesterone | 88 | Washing solution #3 | 0 |
| Progesterone | 88 | Washing solution #4 | 0 |
| Progesterone | 80 | Washing solution #1 | 2.1841 |
| Progesterone | 80 | Washing solution #2 | 0 |
| Progesterone | 80 | Washing solution #3 | 0 |
| Progesterone | 80 | Washing solution #4 | 0 |
| Progesterone | 86 | Washing solution #1 | 2.2352 |
| Progesterone | 86 | Washing solution #2 | 0 |
| Progesterone | 86 | Washing solution #3 | 0 |
| Progesterone | 86 | Washing solution #4 | 0 |

*Table 5: HPLC-MS/MS compound-dependent parameters for the different steroids. RT, retention time, CV, cone voltage, CE, collision energy*

| Analyte | RT (min) | CV  (V) | Transition 1  (quantifier) | CE 1  (V) | Transition 2  (qualifier) | CE 2  (V) |
| --- | --- | --- | --- | --- | --- | --- |
| Cortisol | 3.56 | 35 | 363.2 → 121.1 | 25 | - | - |
| Cortisol-d4 | 3.55 | 30 | 367.2 → 121.1 | 25 | - | - |
| Cortisone | 3.63 | 25 | 361.2 → 163.1 | 22 | - | - |
| 11-KT | 3.97 | 40 | 303.2 → 121.1 | 20 | 303.2 → 259.1 | 22 |
| Corticosterone | 4.43 | 25 | 347.2 → 121.1 | 23 | - | - |
| Corticosterone-d8 | 4.38 | 30 | 355.2 → 125.1 | 25 | - | - |
| Estradiol | 5.19 | 25 | 255.2 → 159.1 | 18 | - | - |
| Estradiol-d4 | 5.17 | 25 | 259.2 → 161.1 | 18 | - | - |
| Testosterone | 5.33 | 15 | 289.2 → 97.1 | 22 | 289.2 → 109.1 | 24 |
| Testosterone-d3 | 5.30 | 15 | 292.2 → 97.1 | 22 | - | - |
| Androstenedione | 5.81 | 30 | 287.1 → 109.1 | 23 | 287.1 → 97.1 | 20 |
| Androstenedione-d7 | 5.77 | 30 | 294.2 → 100.1 | 20 | - | - |
| Progesterone | 7.47 | 25 | 315.2 → 97.1 | 21 | 315.2 → 109.1 | 25 |
| Progesterone-d9 | 7.39 | 25 | 324.2 → 100.1 | 21 | - | - |

*Figure 9: Raw values (points) and prediction plots visualizing the significant sex effect in cortisol concentrations, in breeders.*

*Figure 10: Raw values (points: red = females, turquoise = males) and prediction plots visualizing the significant age effect in a) cortisol and b) the significant interaction effect between sex and age in corticosterone concentrations, in breeders.*

*Figure 11: Raw values (points: red = females, turquoise = males) and prediction plots visualizing the significant age effect in a) cortisol and b) corticosterone concentrations, in helpers.*

*Figure 12: Raw values (points: red = females, turquoise = males) and prediction plots visualizing the significant effect of the presence of dependent offspring in the group on corticosterone in breeders and helpers combined.*

*Figure 13: Raw values (points) and prediction plots visualizing the significant sex effect in testosterone concentrations in a) breeders and b) helpers.*

*Figure 14: Raw values (points: red = females, turquoise = males) and prediction plots visualizing the significant effect of the presence of dependent offspring in the group on testosterone in breeders.*

*Figure 15: Raw values (points) and prediction plots visualizing the significant sex effect in progesterone concentrations in helpers.*

*Figure 16: Raw values (points) and prediction plots visualizing the significant sex effect in 11-ketotestosterone concentrations in a) breeders and b) helpers.*

*Figure 17: Ratio of 11-ketotestosterone to testosterone across the age of all individuals, measured in years. Samples positioned above the red dotted line indicate higher levels of 11-ketotestosterone compared to testosterone.*

*Figure 18: Raw values (points: red = females, turquoise = males) and prediction plots visualizing the significant age effect in cortisone in a) breeders and helpers combined, b) in breeders only and c) in helpers only.*

*Figure 19: Raw values (points) and prediction plots visualizing the significant sex effect in cortisone concentrations in a) breeders and helpers and b) breeders only.*

Figure 20: Raw values (points: red = females, turquoise = males) and prediction plots visualizing the significant effect of the presence of dependent offspring in the group on cortisone concentrations in breeders.

***Model Outputs from Linear Mixed Model Analysis of Hormonal Concentrations:***

This section presents the results from the linear mixed models analyzing the fixed effects on hormonal concentrations measured in picograms per milligram of hair. Concentrations were log-transformed using the log1p function (log1p(pg/mg)) to stabilize variance. The models include predictors such as breeding role (breeder or helper), sex, standardized days (days.z), batch number (referred to as ISBL if applicable), and the presence of dependent offspring (dep_off, as well as potential interaction effects of the main effects. Each table reports chi-square values, degrees of freedom, and p-values for each predictor to highlight their statistical significance. Additionally, the model’s adjusted R-squared (R2c), overall chi-square statistic, and p-value are detailed, along with the number of samples and individuals analyzed.

*Table 6*

| **Cortisol, Primary Dataset:**  **Analysis of Deviance Table (Type II Wald chisquare tests)** | | | |
| --- | --- | --- | --- |
|  | | |  |
| **Response: log1p(pg_mg)** | | |  |
|  | **Chisq** | **Df** | **Pr(>Chisq)** |
| **role** | **7.5428** | **1** | **0.0060250** |
| **sex** | **44.5822** | **1** | **2.439e-11** |
| **days.z** | **26.4332** | **1** | **2.728e-07** |
| **ISBL** | **44.1836** | **1** | **2.990e-11** |
| **dep_off** | **1.9692** | **1** | **0.1605378** |
| **role:sex** | **12.7999** | **1** | **0.0003466** |
|  | | | |
| **n _samples_= 101, n _ind_= 44, R^2^_c_= 0.744, Chisq= 63.522, p= 8.64e-12** | | | |

*Table 7*

| **Cortisol, Breeders-Only Dataset:**  **Analysis of Deviance Table (Type II Wald chisquare tests)** | | | |
| --- | --- | --- | --- |
|  | | |  |
| **Response: log1p(pg_mg)** | | |  |
|  | **Chisq** | **Df** | **Pr(>Chisq)** |
| **sex** | **14.6271** | **1** | **0.000131** |
| **days.z** | **4.4325** | **1** | **0.03526** |
| **ISBL** | **31.9639** | **1** | **1.57e-08** |
| **dep_off** | **5.5019** | **1** | **0.018996** |
|  | | | |
| **n _samples_= 104, n _ind_= 37, R^2^_c_= 0.666, Chisq= 40.278, p= 3.792e-08** | | | |

*Table 8*

| **Cortisol, Helpers-Only Dataset:**  **Analysis of Deviance Table (Type II Wald chisquare tests)** | | | |
| --- | --- | --- | --- |
|  | | |  |
| **Response: log1p(pg_mg)** | | |  |
|  | **Chisq** | **Df** | **Pr(>Chisq)** |
| **sex** | **3.5312** | **1** | **0.06022** |
| **days.z** | **30.6249** | **1** | **3.13e-08** |
| **ISBL** | **1.787** | **1** | **1.81e-01** |
|  | | | |
| **n _samples_= 89, n _ind_= 36 , R^2^_c_= 0.484, Chisq= 25.187, p= 1.411e-05** | | | |

*Table 9*

| **Cortisone, Primary Dataset:**  **Analysis of Deviance Table (Type II Wald chisquare tests)** | | | |
| --- | --- | --- | --- |
|  | | |  |
| **Response: log1p(pg_mg)** | | |  |
|  | **Chisq** | **Df** | **Pr(>Chisq)** |
| **role** | **2.1357** | **1** | **0.1439054** |
| **sex** | **8.4160** | **1** | **0.0037193** |
| **days.z** | **13.8182** | **1** | **0.0002014** |
| **ISBL** | **24.1993** | **1** | **8.686e-07** |
| **dep_off** | **1.7743** | **1** | **0.1828450** |
| **role:sex** | **4.3136** | **1** | **0.0378089** |
|  | | | |
| **n _samples_= 101, n _ind_= 44, R^2^_c_= 0.636, Chisq= 34.689, p= 4.95e-06** | | | |

*Table 10*

| **Cortisone, Breeders-Only Dataset:**  **Analysis of Deviance Table (Type II Wald chisquare tests)** | | | |
| --- | --- | --- | --- |
|  | | |  |
| **Response: log1p(pg_mg)** | | |  |
|  | **Chisq** | **Df** | **Pr(>Chisq)** |
| **sex** | **4.9719** | **1** | **0.025762** |
| **days.z** | **6.8689** | **1** | **8.77e-03** |
| **dep_off** | **5.0234** | **1** | **2.50e-02** |
| **ISBL** | **24.9458** | **1** | **5.90e-07** |
|  | | | |
| **n _samples_= 104, n _ind_= 37, R^2^_c_= 0.539, Chisq= 30.290, p= 4.273e-06** | | | |

*Table 11*

| **Cortisone, Helpers-Only Dataset:**  **Analysis of Deviance Table (Type II Wald chisquare tests)** | | | |
| --- | --- | --- | --- |
|  | | |  |
| **Response: log1p(pg_mg)** | | |  |
|  | **Chisq** | **Df** | **Pr(>Chisq)** |
| **sex** | **0.1888** | **1** | **0.6639** |
| **days.z** | **26.8575** | **1** | **2.19e-07** |
| **ISBL** | **0.0809** | **1** | **0.7661** |
|  | | | |
| **n _samples_= 89, n _ind_= 36, R^2^_c_= 0.484, Chisq= 21.188, p= 9.623e-05** | | | |

*Table 12*

| **Corticosterone, Primary Dataset:**  **Analysis of Deviance Table (Type II Wald chisquare tests)** | | | |
| --- | --- | --- | --- |
|  | | |  |
| **Response: log1p(pg_mg)** | | |  |
|  | **Chisq** | **Df** | **Pr(>Chisq)** |
| **role** | **5.9121** | **1** | **0.015038** |
| **sex** | **30.4535** | **1** | **3.420e-08** |
| **days.z** | **10.3992** | **1** | **0.001261** |
| **ISBL** | **58.6850** | **1** | **1.851e-14** |
| **dep_off** | **4.2304** | **1** | **0.039707** |
| **role:sex** | **7.2806** | **1** | **0.006970** |
|  | | | |
| **n _samples_= 101, n _ind_= 44, R^2^_c_= 0.796, Chisq= 66.92, p= 1.747e-12** | | | |

*Table 13*

| **Corticosterone, Breeders-Only Dataset:**  **Analysis of Deviance Table (Type II Wald chisquare tests)** | | | |
| --- | --- | --- | --- |
|  | | |  |
| **Response: log1p(pg_mg)** | | |  |
|  | **Chisq** | **Df** | **Pr(>Chisq)** |
| **sex** | **25.2123** | **1** | **5.14e-07** |
| **days.z** | **3.8614** | **1** | **4.94e-02** |
| **dep_off** | **10.3301** | **1** | **1.31e-03** |
| **ISBL** | **54.1119** | **1** | **1.89e-13** |
| **sex:days.z** | **8.4631** | **1** | **0.003624** |
|  | | | |
| **n _samples_= 104, n _ind_= 37, R^2^_c_= 0.759, Chisq= 65.21, p= 1.014e-12** | | | |

*Table 14*

| **Corticosterone, Helpers-Only Dataset:**  **Analysis of Deviance Table (Type II Wald chisquare tests)** | | | |
| --- | --- | --- | --- |
|  | | |  |
| **Response: log1p(pg_mg)** | | |  |
|  | **Chisq** | **Df** | **Pr(>Chisq)** |
| **sex** | **3.5312** | **1** | **6.02e-02** |
| **days.z** | **30.6249** | **1** | **3.13e-08** |
| **ISBL** | **1.787** | **1** | **1.81e-01** |
|  | | | |
| **n _samples_= 89, n _ind_= 36, R^2^_c_= 0.485, Chisq= 17.259, p= 0.0006251** | | | |

*Table 15*

| **Testosterone, Primary Dataset:**  **Analysis of Deviance Table (Type II Wald chisquare tests)** | | | |
| --- | --- | --- | --- |
|  | | |  |
| **Response: log1p(pg_mg)** | | |  |
|  | **Chisq** | **Df** | **Pr(>Chisq)** |
| **role** | **0.7962** | **1** | **0.37225** |
| **sex** | **5.1824** | **1** | **0.02282** |
| **days.z** | **0.2814** | **1** | **0.59578** |
| **ISBL** | **5.3831** | **1** | **0.02033** |
| **dep_off** | **5.7052** | **1** | **0.01691** |
|  | | | |
| **n _samples_= 101, n _ind_= 44, R^2^_c_= 0.586, Chisq= 14.143, p= 0.01472** | | | |

*Table 16*

| **Testosterone, Breeders-Only Dataset:**  **Analysis of Deviance Table (Type II Wald chisquare tests)** | | | |
| --- | --- | --- | --- |
|  | | |  |
| **Response: log1p(pg_mg)** | | |  |
|  | **Chisq** | **Df** | **Pr(>Chisq)** |
| **sex** | **11.9849** | **1** | **5.36e-04** |
| **ns(days.z, df = 4)** | **3.5047** | **4** | **0.477** |
| **dep_off** | **8.9141** | **1** | **2.83e-03** |
| **ISBL** | **6.1957** | **1** | **1.28e-02** |
|  | | | |
| **n _samples_= 104, n _ind_= 37, R^2^_c_= 0.484, Chisq= 24.500, p= 0.00093** | | | |

*Table 17*

| **Testosterone, Helpers-Only Dataset:**  **Analysis of Deviance Table (Type II Wald chisquare tests)** | | | |
| --- | --- | --- | --- |
|  | | |  |
| **Response: log1p(pg_mg)** | | |  |
|  | **Chisq** | **Df** | **Pr(>Chisq)** |
| **sex** | **12.7754** | **1** | **3.51e-04** |
| **ns(days.z, df = 4)** | **162.8679** | **4** | **< 2.2e-16** |
| **ISBL** | **0.1239** | **1** | **7.25e-01** |
|  | | | |
| **n _samples_= 89, n _ind_= 36, R^2^_c_= 0.773, Chisq= 91.767, p= < 2.2e-16** | | | |

*Table 18*

| **Androstenedione, Helpers-Only Dataset:**  **Analysis of Deviance Table (Type II Wald chisquare tests)** | | | |
| --- | --- | --- | --- |
|  | | |  |
| **Response: log1p(pg_mg)** | | |  |
|  | **Chisq** | **Df** | **Pr(>Chisq)** |
| **sex** | **2.3501** | **1** | **1.25e-01** |
| **ns(days.z, df = 4)** | **68.6563** | **4** | **4.36e-14** |
| **ISBL** | **0.0011** | **1** | **9.74e-01** |
|  | | | |
| **n _samples_= 85, n _ind_= 36, R^2^_c_= 0.763, Chisq= 58.73, p= 8.15e-11** | | | |

*Table 19*

| **11-Ketotestosterone, Primary Dataset:**  **Analysis of Deviance Table (Type II Wald chisquare tests)** | | | |
| --- | --- | --- | --- |
|  | | |  |
| **Response: log1p(pg_mg)** | | |  |
|  | **Chisq** | **Df** | **Pr(>Chisq)** |
| **role** | **0.9590** | **1** | **0.3274** |
| **sex** | **41.0061** | **1** | **1.518e-10** |
| **days.z** | **0.2036** | **1** | **0.6518** |
| **ISBL** | **0.7557** | **1** | **0.3847** |
| **dep_off** | **1.9372** | **1** | **0.1640** |
|  | | | |
| **n _samples_= 101, n _ind_= 44, R^2^_c_= 0.575, Chisq= 32.197, p= 5.431e-06** | | | |

*Table 20*

| **11-Ketotestosterone, Breeders-Only Dataset:**  **Analysis of Deviance Table (Type II Wald chisquare tests)** | | | |
| --- | --- | --- | --- |
|  | | |  |
| **Response: log1p(pg_mg)** | | |  |
|  | **Chisq** | **Df** | **Pr(>Chisq)** |
| **sex** | **34.8589** | **1** | **3.55e-09** |
| **ns(days.z, df = 4)** | **7.032** | **4** | **1.34e-01** |
| **dep_off** | **3.1929** | **1** | **7.40e-02** |
| **ISBL** | **0.3505** | **1** | **5.54e-01** |
|  | | | |
| **n _samples_= 104, n _ind_= 37, R^2^_c_= 0.457, Chisq= 27.456, p= 2.758e-04** | | | |

*Table 21*

| **11-Ketotestosterone, Helpers-Only Dataset:**  **Analysis of Deviance Table (Type II Wald chisquare tests)** | | | |
| --- | --- | --- | --- |
|  | | |  |
| **Response: log1p(pg_mg)** | | |  |
|  | **Chisq** | **Df** | **Pr(>Chisq)** |
| **sex** | **23.0316** | **1** | **1.59e-06** |
| **ns(days.z, df = 4)** | **23.668** | **4** | **9.31e-05** |
| **ISBL** | **2.3393** | **1** | **1.26e-01** |
|  | | | |
| **n _samples_= 89, n _ind_= 36, R^2^_c_= 0.625, Chisq= 48.178, p= 1.089e-08** | | | |

*Table 22*

| **Estradiol, Primary Dataset:**  **Analysis of Deviance Table (Type II Wald chisquare tests)** | | | |
| --- | --- | --- | --- |
|  | | |  |
| **Response: log1p(pg_mg)** | | |  |
|  | **Chisq** | **Df** | **Pr(>Chisq)** |
| **role** | **1.7713** | **1** | **0.183224** |
| **sex** | **1.7990** | **1** | **0.179833** |
| **days.z** | **0.1711** | **1** | **0.679162** |
| **ISBL** | **8.5875** | **1** | **0.003385** |
| **dep_off** | **0.4794** | **1** | **0.488702** |
|  | | | |
| **n _samples_= 101, n _ind_= 44, R^2^_c_= 0.317, Chisq= 13.062, p= 0.02281** | | | |

*Table 23*

| **Estradiol, Breeders-Only Dataset:**  **Analysis of Deviance Table (Type II Wald chisquare tests)** | | | |
| --- | --- | --- | --- |
|  | | |  |
| **Response: log1p(pg_mg)** | | |  |
|  | **Chisq** | **Df** | **Pr(>Chisq)** |
| **sex** | **12.2634** | **1** | **4.62e-04** |
| **ns(days.z, df = 4)** | **5.0167** | **4** | **2.86e-01** |
| **dep_off** | **0.12** | **1** | **7.29e-01** |
| **ISBL** | **5.7372** | **1** | **1.66e-02** |
|  | | | |
| **n _samples_= 102, n _ind_= 37, R^2^_c_= 0.515, Chisq= 19.926, p= 0.005731** | | | |

*Table 24*

| **Estradiol, Helpers-Only Dataset:**  **Analysis of Deviance Table (Type II Wald chisquare tests)** | | | |
| --- | --- | --- | --- |
|  | | |  |
| **Response: log1p(pg_mg)** | | |  |
|  | **Chisq** | **Df** | **Pr(>Chisq)** |
| **sex** | **0.4334** | **1** | **5.10e-01** |
| **ns(days.z, df = 4)** | **9.5172** | **4** | **4.94e-02** |
| **ISBL** | **13.8843** | **1** | **1.94e-04** |
|  | | | |
| **n _samples_= 89, n _ind_= 36, R^2^_c_= 0.377, Chisq= 18.942, p= 0.004262** | | | |

*Table 25*

| **Progesterone, Primary Dataset:**  **Analysis of Deviance Table (Type II Wald chisquare tests)** | | | |
| --- | --- | --- | --- |
|  | | |  |
| **Response: log1p(pg_mg)** | | |  |
|  | **Chisq** | **Df** | **Pr(>Chisq)** |
| **role** | **7.4328** | **1** | **0.006405** |
| **sex** | **152.4111** | **1** | **< 2.2e-16** |
| **days.z** | **0.3377** | **1** | **0.561154** |
| **ISBL** | **4.7792** | **1** | **0.028805** |
| **dep_off** | **2.7052** | **1** | **0.100021** |
| **role:sex** | **9.3238** | **1** | **0.002262** |
|  | | | |
| **n _samples_= 101, n _ind_= 44, R^2^_c_= 0.953, Chisq= 78.319, p= 7.946e-15** | | | |

*Table 26*

| **Progesterone, Breeders-Only Dataset:**  **Analysis of Deviance Table (Type II Wald chisquare tests)** | | | |
| --- | --- | --- | --- |
|  | | |  |
| **Response: log1p(pg_mg)** | | |  |
|  | **Chisq** | **Df** | **Pr(>Chisq)** |
| **sex** | **1210.8511** | **1** | **< 2.2e-16** |
| **ns(days.z, df = 4)** | **8.9305** | **4** | **6.29e-02** |
| **dep_off** | **3.5671** | **1** | **5.89e-02** |
| **ISBL** | **3.5114** | **1** | **6.09e-02** |
| **sex:ns(days.z, df = 4)** | **22.0249** | **4** | **0.0001981** |
|  | | | |
| **n _samples_= 104, n _ind_= 37, R^2^_c_= 0.967, Chisq= 128.14, p= < 2.2e-16** | | | |

*Table 27*

| **Progesterone, Helpers-Only Dataset:**  **Analysis of Deviance Table (Type II Wald chisquare tests)** | | | |
| --- | --- | --- | --- |
|  | | |  |
| **Response: log1p(pg_mg)** | | |  |
|  | **Chisq** | **Df** | **Pr(>Chisq)** |
| **sex** | **19.0143** | **1** | **1.30e-05** |
| **ns(days.z, df = 4)** | **32.6244** | **4** | **1.43e-06** |
| **ISBL** | **4.9221** | **1** | **2.65e-02** |
|  | | | |
| **n _samples_= 89, n _ind_= 36, R^2^_c_= 0.803, Chisq= 56.854, p= 1.956e-10** | | | |

*Figure 21: Pearson Correlation Matrix: Numbers and colors represent Pearson correlation coefficients ranging from -1 (perfect negative correlation) to +1 (perfect positive correlation). Values that are crossed out indicate coefficients that are above the significance threshold (p > 0.05) and are not considered statistically significant.*
